# Rate-Engineered Plasmon-Enhanced Fluorescence for Real-Time Microsecond Dynamics of Single Biomolecules

**DOI:** 10.1021/acs.nanolett.4c03220

**Published:** 2024-09-09

**Authors:** Sjoerd W. Nooteboom, Kasper R. Okholm, Vincenzo Lamberti, Bas Oomen, Duncan S. Sutherland, Peter Zijlstra

**Affiliations:** † Department of Applied Physics and Science Education, Eindhoven University of Technology, 5600 MB Eindhoven, The Netherlands; ‡ Institute for Complex Molecular Systems, Eindhoven University of Technology, 5600 MB Eindhoven, The Netherlands; § Interdisciplinary Nanoscience Center, Aarhus University, 8000 Aarhus C, Denmark; ∥ The Centre for Cellular Signal Patterns (CELLPAT), 8000 Aarhus C, Denmark

**Keywords:** nanoscale sensing, plasmon-enhanced fluorescence, single-molecule detection, single gold nanoparticles

## Abstract

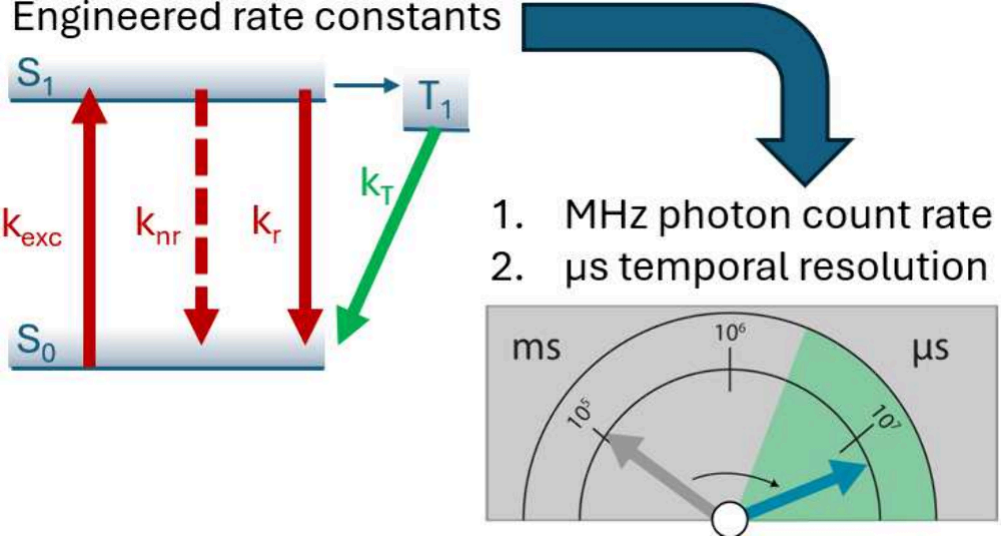

Single-molecule fluorescence has revealed a wealth of biochemical processes but does not give access to submillisecond dynamics involved in transient interactions and molecular dynamics. Here we overcome this bottleneck and demonstrate record-high photon count rates of >10^7^ photons/s from single plasmon-enhanced fluorophores. This is achieved by combining two conceptual novelties: first, we balance the excitation and decay rate enhancements by the antenna’s volume, resulting in maximum fluorescence intensity. Second, we enhance the triplet decay rate using a multicomponent surface chemistry that minimizes microsecond blinking. We demonstrate applications to two exemplary molecular processes: we first reveal transient encounters and hybridization of DNA with a 1 μs temporal resolution. Second, we exploit the field gradient around the nanoparticle as a molecular ruler to reveal microsecond intramolecular dynamics of multivalent complexes. Our results pave the way toward real-time microsecond studies of biochemical processes using an implementation compatible with existing single-molecule fluorescence methods.

Biomolecules such as nucleic acids and proteins form the functional basis of all living organisms. Their functionality originates from dynamic processes, such as folding, conformational changes, and intermolecular interactions. Prime examples are the conformational changes and interaction dynamics of enzymes,^1,2^ intrinsically disordered proteins (IDPs),^3,4^ nucleic acids,^5,6^ and cellular receptors.^7^ Critically, many biomolecular interactions are multivalent, where a stronger bond is accomplished by combining multiple low-affinity (and thus dynamic) bonds.^8^ All these processes span a very broad range of time scales from picoseconds to hours.^9,10^ A combination of molecular dynamics simulations and ensemble-averaged studies has revealed that such fast dynamics are crucial for the biological function, while misregulation of the dynamics can lead to disease due to e.g. misfolding^11^ or aberrant interaction thermodynamics.^12^ Overall, understanding the mechanisms of biomolecular dynamics on all relevant time scales is key to progress in molecular biology and medicine.

Biomolecular dynamics are often studied in solution, where fluorescence correlation spectroscopy resolves dynamics down to nanoseconds by averaging over many single-molecule passages through a focused laser beam. However, such ensemble-averaging methods hide underlying static and dynamic heterogeneity despite its importance for function and disease.^13,14^ Real-time single-molecule fluorescence of immobilized biomolecules directly reveals dynamics and heterogeneity in time and has provided a wealth of information on molecular mechanisms of e.g. DNA^15,16^ and proteins.^14,17−20^ However, the brightness of a fluorophore saturates at a photon count rate (CR) of about 10^5^ photons/s, so sub-ms time scales can again only be obtained by averaging over hundreds to thousands of single molecules to obtain a sufficient signal-to-noise ratio.^21,22^

Recent experiments focused on bringing single-molecule studies into the microsecond domain. Label-free detection based on plasmonic nanopores,^23^ Fabry–Pérot microcavities,^24^ or interferometric microscopy^25^ has probed biomolecular diffusion. Label-free techniques usually do not suffer from signal saturation, but their lack of chemical specificity does not allow for probing specific biomolecular processes. Another study employed polycrystalline plasmonic nanoapertures to enhance single-molecule fluorescence signals.^26^ This pioneering study showed the promise of using nanoplasmonic approaches to boost fluorophore brightness, but the application was limited to probing diffusion. Very recently, fast interactions between biomolecules were studied using plasmon-enhanced Förster resonance energy transfer^27^ using complex assemblies of nanoparticle dimers on a DNA origami. Experimental access to biomolecular dynamics therefore remains challenging yet critical to gain insight into fast processes.

Here, we use individual single-crystalline gold nanoparticles to further push the fluorescence intensity and demonstrate real-time probing of molecular processes with a temporal resolution of 1 μs. This is achieved by combining two conceptual novelties: first, we study by experiment and simulation the effect of antenna volume on the excitation and decay rates of a plasmon-coupled fluorophore. We find an optimum antenna volume that balances the different rate enhancements, resulting in maximized fluorescence intensity. Second, we introduce a multicomponent surface chemistry that enhances the triplet decay rate and thereby minimizes microsecond blinking. Such rate-engineering provides a continuous stream of more than 10^7^ photons/s for a single organic dye, improving more than 2 orders of magnitude over previous reports.^28,29^ We then demonstrate two applications: first, we monitor single-molecule DNA diffusion followed by association and dissociation on microsecond time scales. Second, we exploit the gradient in fluorescence enhancement around the nanoparticle as a ruler to reveal the microsecond molecular dynamics of a multivalent complex. The presented approach is compatible with any commercial fluorescence microscope, does not require complex nanoparticle assembly, and therefore expands the utility of single-molecule fluorescence toward microsecond processes and beyond.

We use single gold nanorods (AuNRs) as the core geometry since they can be synthesized colloidally with high purity in single-crystalline form.^30,31^ The latter is crucial, as the elimination of defects improves the fluorescence enhancement compared to lithographic structures due to reduced plasmon dephasing.^32^ In addition, their localized surface plasmon resonance (LSPR) occurs away from the interband transitions, further reducing plasmon dephasing.^33,34^ The particles were immobilized on a glass slide at low density and functionalized with thiolated single-stranded DNA receptors (see Figure 1a,b and Supporting Information (SI) section S1). The sample was inserted in a flow cell and mounted on a standard fluorescence microscope with a 637 nm laser excitation. Ligand (monovalent or multivalent) labeled with Atto655 was introduced in the flow cell, resulting in transient single-molecule interactions with the receptor strands on the particle. The single-molecule interactions were detected on a camera for millisecond temporal resolution or on a single-photon counting avalanche photodiode (SPAD) for microsecond measurements (see SI section S1 for experimental details). Figure 1c contains an exemplary time trace showing short-lived bursts of plasmon-enhanced fluorescence due to 9 nucleotide (nt) ligand interactions that are superimposed on a stable baseline due to the particle’s one-photon luminescence.^28,35^

**Figure 1:**
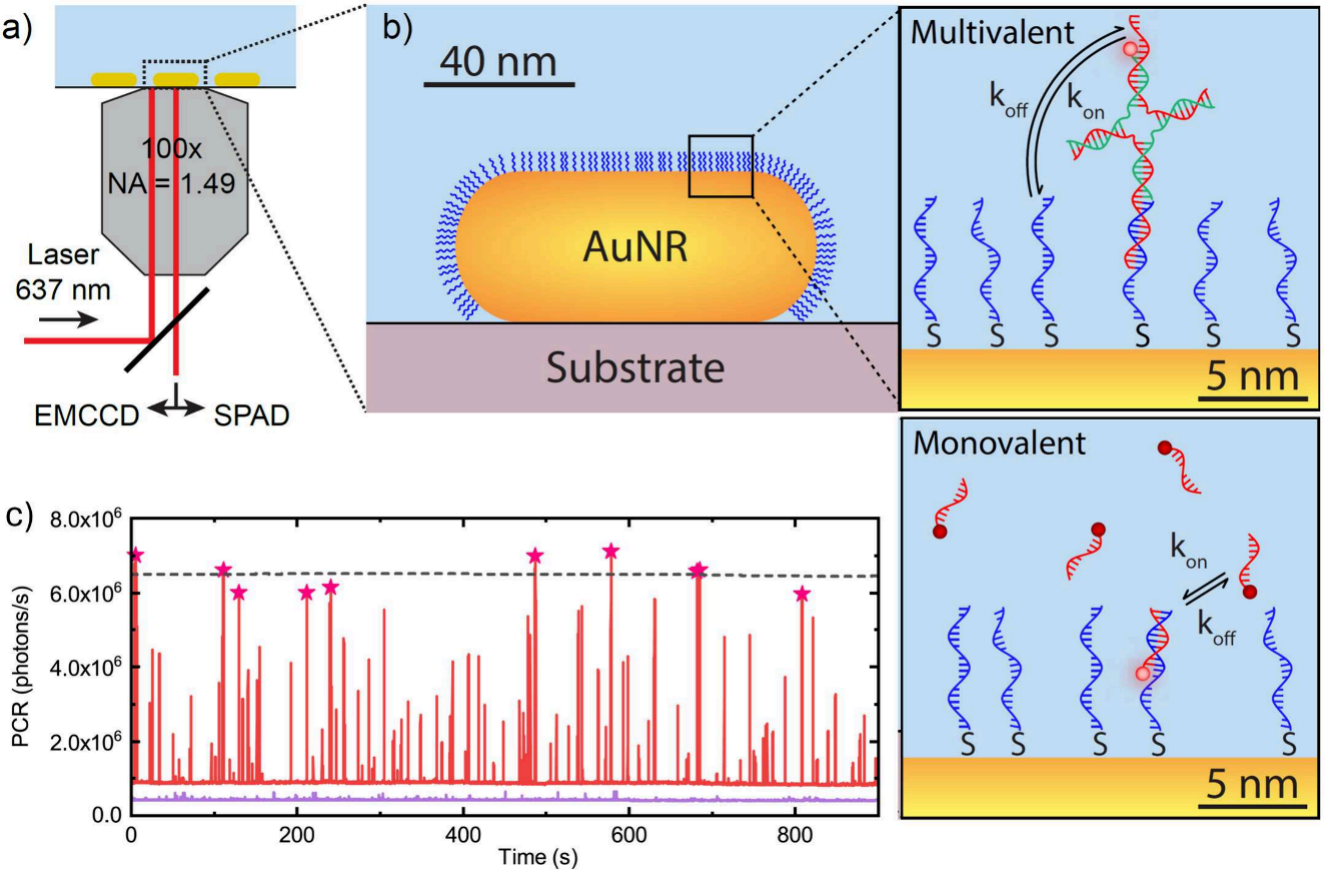
(a) Schematic illustration of the setup used in this work. The sample consisted of AuNRs that were spin-coated on a glass coverslip at low density and mounted in a flow cell. Single-molecule fluorescence signals were detected on an electron-multiplying charge-coupled device (EMCCD) for millisecond dynamics or on a SPAD for microsecond dynamics. (b) The gold particles are functionalized with thiolated single-stranded DNA receptors (blue). The introduction of monovalent or bivalent ligands results in transient single-molecule interactions with characteristic rate constants *k*_on_ and *k*_off_. (c) A section of a fluorescence time trace of monovalent ssDNA ligands transiently interacting with a single AuNR recorded on the camera (red) compared to the background signal (blue). The brightest events are highlighted by red stars; the dashed line indicates their average photon CR.

To achieve a continuous stream of 10^7^ photons per second from a fluorescently labeled ligand, we address two key aspects. First, we balance the enhancements of the excitation, radiative, and nonradiative rates. To this end we performed numerical simulations where we assessed the rate enhancements for a series of four nanorods with dimensions of 10 × 24, 25 × 56, 40 × 82, and 70 × 114 nm^2^. Previous correlative microscopy has indicated that (for nanorods) simulations of the average size and shape of the particles in the distribution are a good representation and enable quantitative analysis.^36,37^ We therefore use the prototypical spherically capped cylinder as the core geometry in our simulations. The dimensions were chosen such that the width of the particle matches manufacturer specifications, while the length was chosen to obtain maximum fluorescence enhancement (see SI S1 and S2 for details and results). The simulations indicate that the excitation rate enhancement at 637 nm peaks for a diameter of 25 nm, after which it decreases because radiation damping broadens the plasmon resonance. The simulations further indicate that the radiative rate enhancement for ATTO655 is nearly independent of particle volume for the larger particles. However, the nonradiative rate enhancement strongly reduces with particle volume. This can be understood by considering the radiative efficiency or albedo of the particle that is often calculated as the ratio between scattering and extinction cross sections.^38^ The albedo essentially represents the quantum yield of the antenna and peaks at ∼40% for a diameter of 40 nm, thereby providing the highest overall photon CR.

We verified this experimentally in Figure 2a by recording time traces and averaging the 10 brightest events per particle (see the dotted line in Figure 1c). For every diameter, we observe a distribution of values for the photon CR due to the varying locations of each particle in the Gaussian excitation beam (affecting the local excitation intensity) and the heterogeneous LSPR wavelengths (affecting the spectral overlap with the excitation and emission wavelengths). Our experiments confirm the simulations, indicating that 40 nm particles exhibit the strongest photon CR approaching 10^7^ photons per second.

**Figure 2:**
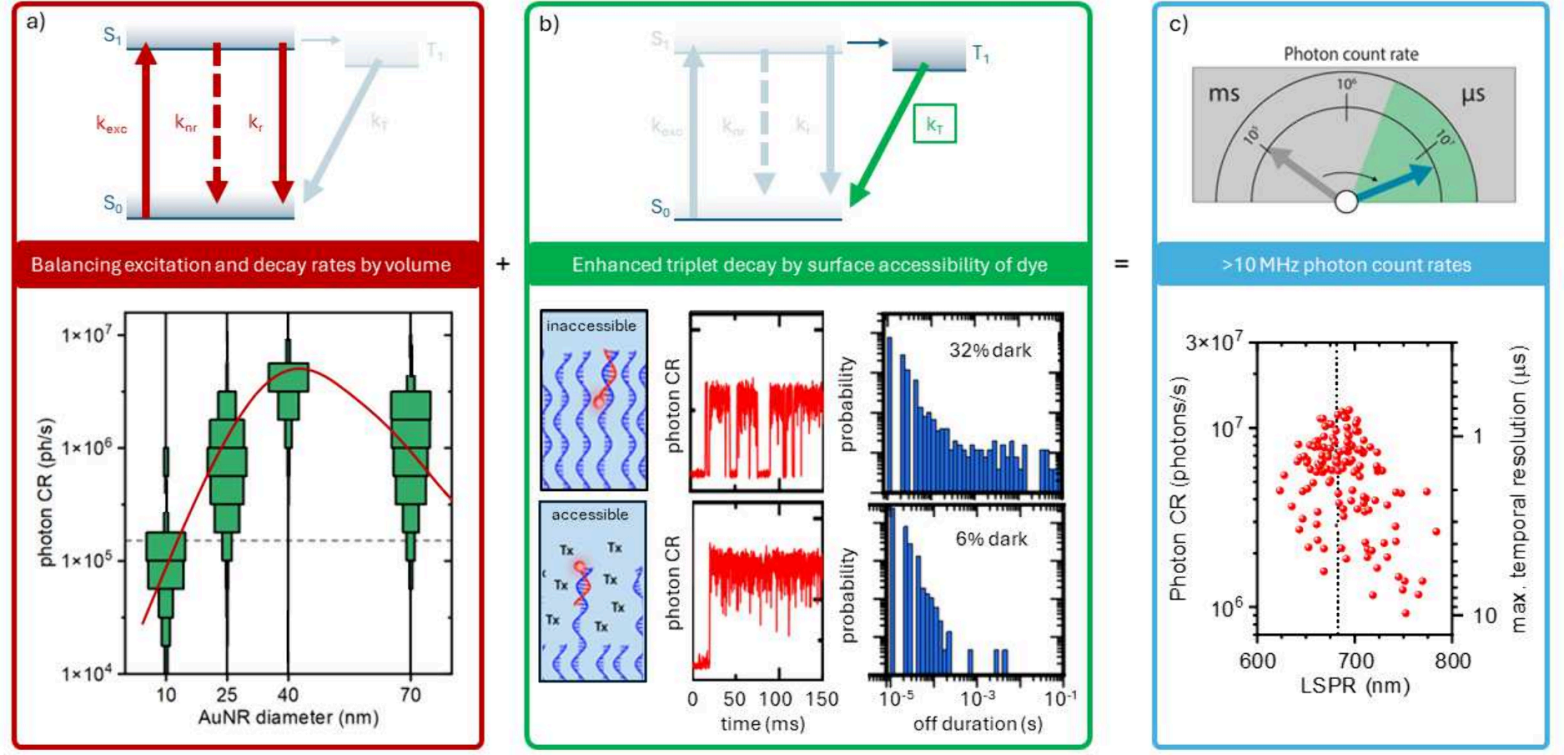
Rate engineering to achieve a microsecond temporal resolution. (a) Distribution of obtained photon CR values of the top 10 bursts for each single particle, as a function of particle volume (indicated by its average diameter). Tuning the nanoparticle volume modulates the balance between excitation, radiative, and nonradiative rate enhancement. All samples have an ensemble-average plasmon wavelength of 650 nm. The red line is a guide to the eye while the dashed line indicates the non-enhanced PCR. (b) Enhancing the triplet decay rate by the introduction of a multicomponent surface coating that maximizes the accessibility to triplet quenchers. The red traces show exemplary single-molecule binding events with a 100 μs binning time, showing clear blinking events. The histograms show the number of blinks per second as a function of off-blink duration under the two different conditions, obtained from 10 μs binned time traces. The percentages indicate the fraction of time the dye spends in the dark state. (c) Measured photon CR under optimized conditions as a function of longitudinal plasmon resonance (LSPR) for AuNRs with a diameter of 40 nm. The vertical dashed line indicates the emission peak of the dye. The right axis indicates the maximum achievable temporal resolution for a signal-to-noise ratio of 3 with a typical dark-count rate of 100 counts per second.

Second, we sought further improvement by minimizing short-lived dark states; see Figure 2b. The obtained microsecond temporal resolution allows us to directly quantify microsecond blinking dynamics in real time. The middle column in Figure 2b shows the start of a typical event for a 9 nt ligand pointing toward the nanoparticle surface. Clear dark states are observed with durations ranging from 10 μs to >10 ms, while the dye spends on average 32% of the time in a dark state.

Upon addition of Trolox, a well-known triplet state quencher, the observed off-blinks become markedly shorter, but the dye still spends >10% of its time in a dark state (see SI section S3). To improve the efficiency of Trolox, we developed a multicomponent surface coating consisting of a mixture of ssDNA receptors and short ssDNA spacers to maximize the accessibility of the dye to solution-phase Trolox. In this case, the improved collision rate between Trolox and the fluorophore eliminates off-blinks lasting longer than 200 μs, while the dye (on average) spends only 6% of its time in a dark state. Note that the molecular association rate of ssDNA probes also depends on the number of strands per particle and their accessibility. Previous work^39^ showed that dilution with a short spacer strand reduces the number of docking strands per particle but simultaneously increases their accessibility. As a result, the overall association rate per particle is nearly unaffected by inclusion of the spacer strand.

The sum of both aspects discussed above results in a final photon CR well above 10^7^ photons/s for many AuNRs, 2 orders of magnitude higher than in our previous work.^28,29^ As shown in Figure 2c, the highest photon CR is achieved for particles with a plasmon that is resonant with the dye’s emission peak, implying that we operate close to the saturation point of Atto643. Overall the synergistic optimization of decay rates by nanoantenna volume and surface chemistry results in a continuous photon CR well over 10^7^ photons per second.

We apply this ultrabright photoemission to two exemplary biomolecular systems. First, we probe transient single-molecule interactions on microsecond time scales, shown in Figure 3a. For a 1 μs binning time, the fluorescence intensities of (1–2) × 10^7^ photons/s correspond to 10–20 photons per bin. To ensure we interrogate single particles rather than clusters, we perform white-light spectroscopy (see SI section S4). For ssDNA hybridization of a 5 nt strand we observe clear binding events with a duration of 10–100 μs. In addition, the signal exhibits microsecond fluctuations while the ssDNA is bound. These are unlikely to be caused by blinking because (with a few exceptions) the fluctuations do not drop to baseline level. We hypothesize that the receptor strand itself undergoes Brownian motion, thereby modulating the distance and orientation of the dye with respect to the AuNR. More examples of events for different ligands are shown in the SI section S5.

**Figure 3:**
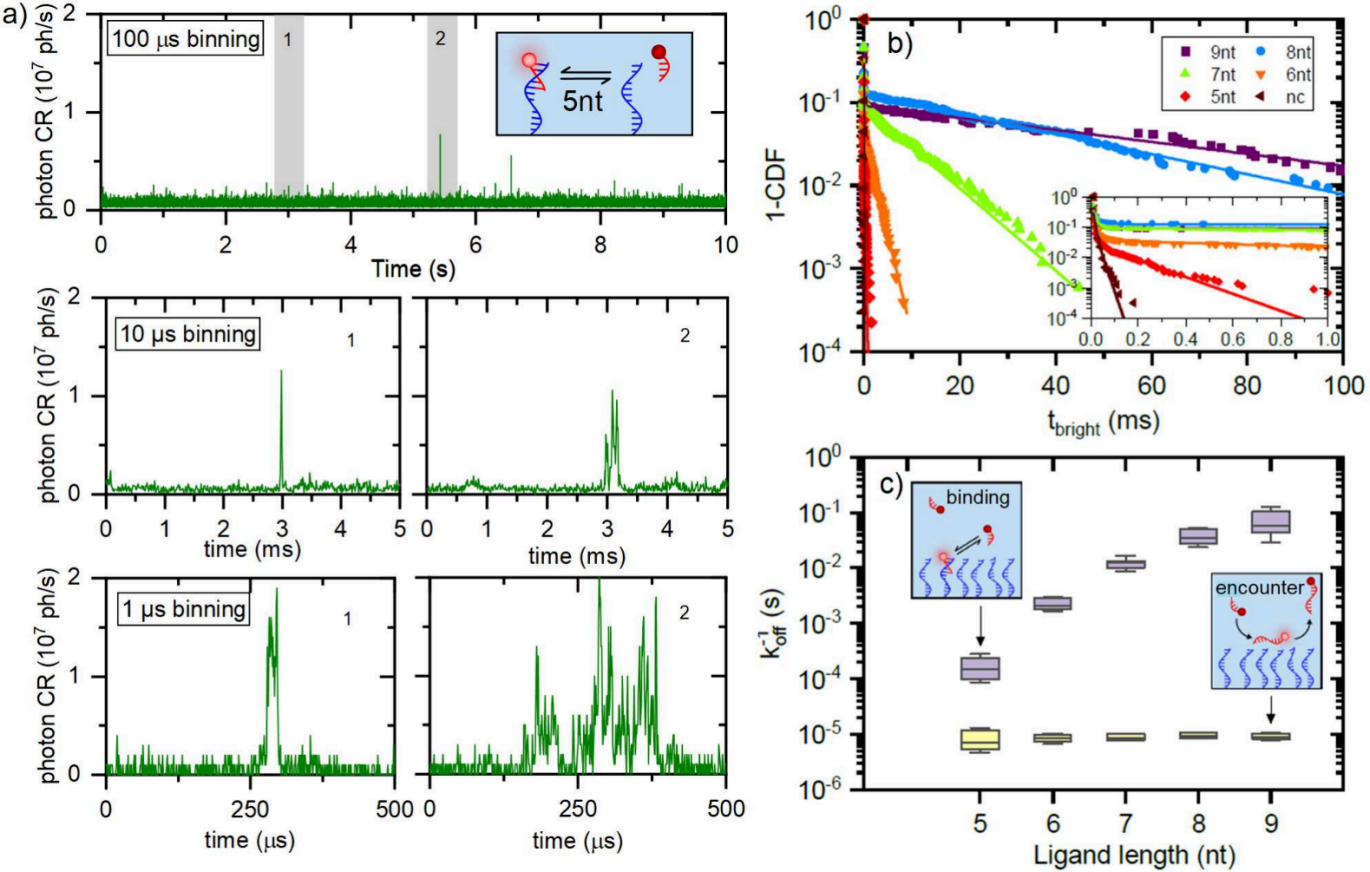
(a) Timetraces for 5 nt ligands with increasingly shortened binning time. (b) Bright time distributions extracted from time traces of a single particle with ligands of different lengths, as well as the noncomplementary (nc) control. The value for *t*_bright_ indicates the duration of individual events, of which 600–4500 were measured for each condition. Inset: zoomed-in to the first millisecond of the same data. (c) Characteristic times obtained from double-exponential fits to the distributions in (b): yellow, fast component; purple, slow component. Boxes indicate 25–75 percentiles with the median line; whiskers indicate 10–90 percentiles. For each ligand, between 12 and 20 AuNRs were measured.

The real-time observation of DNA binding events down to microsecond time scales allows us to straightforwardly quantify the bound-state lifetime even for ultralow-affinity interactions. We quantify the distribution of bound-state lifetimes for a range of ligand lengths (Figure 3b and SI sections S1 and S5). We observe double-exponential behavior, and therefore, the fits of the cumulative distribution function (CDF) yield two distinct dissociation rates *k*_off_. The slow component represents biomolecular binding and changes by nearly 3 orders of magnitude from 60 ms (median) for 9 nt to 150 μs for 5 nt ligands. Note that the bound-state lifetime is sequence and temperature dependent and therefore varies from particle to particle.^40^ The change in average bound-state lifetime from 5 nt to 6 nt is an order of magnitude due to the addition of a C/G pair, whereas the relative changes between the longer ligands are factors of 2–5 due to the addition of lower-affinity A/T pairs.^41,42^

The fast component represents approximately 90% of the events with a nearly constant duration of 10 μs across all ligand lengths, including noncomplementary strands. Estimating a hydrodynamic radius of 1 nm for the ligands,^43^ we find a diffusion coefficient of 260 μm^2^/s at 300 K, so the ligands would diffuse approximately 50 nm, comparable to the size of the AuNR, in 10 μs. The fast component thus corresponds to diffusion of the ligand through the near-field, followed by a 10% probability of hybridization.

Beyond monovalency, multivalency is abundant in nature, wherein multiple low-affinity binding sites are combined to increase the overall avidity. Examples are viruses and antibodies that interact with cells and antigens, respectively. Multivalency has been studied at the single-molecule level, where the avidity is quantified by measuring the overall bound-state lifetime of a multivalent complex.^29,44−46^ However, the underlying monovalent kinetics that underpin the avidity remain elusive because the low affinity of each site results in very fast dynamics and requires submillisecond temporal resolution to resolve.

Therefore, we exploit the achieved microsecond temporal resolution to study for the first time the microsecond dynamics of a single multivalent complex, a Holliday junction^5^ (HJ; see Figure 1b for the design and SI Table S1 for the sequences). The HJ exhibits two binding sites: one 12 nt site that keeps the construct bound to the particle surface for a few seconds and another low-affinity site that transiently interacts with receptor strands on the particle surface. The low-affinity site of the HJ is labeled with a fluorophore that generates a plasmon-enhanced fluorescence signal. The nanoparticle–dye pair now acts as a molecular ruler, whereby the gradient in the fluorescence enhancement near the particle surface transduces distance changes to intensity modulations. Note that in contrast to Förster resonance energy transfer, this approach only requires a single dye label.

We employ three different designs that all exhibit one arm with a 12 nt binding site, while the second arm has no binding site (HJ0), or an 8 nt (HJ8) or 6 nt (HJ6) site (Figure 4, left column). For HJ6 and HJ8 the presence of transient interactions of the second binding site with receptors on the particle surface will change the dye-AuNR distance from approximately 13 nm (monovalent state) to approximately 6 nm (bivalent state; see SI sections S6 and S7). This results in rapid changes in photon CR because the dye’s position is modulated in the decaying near-field of the AuNR.

**Figure 4:**
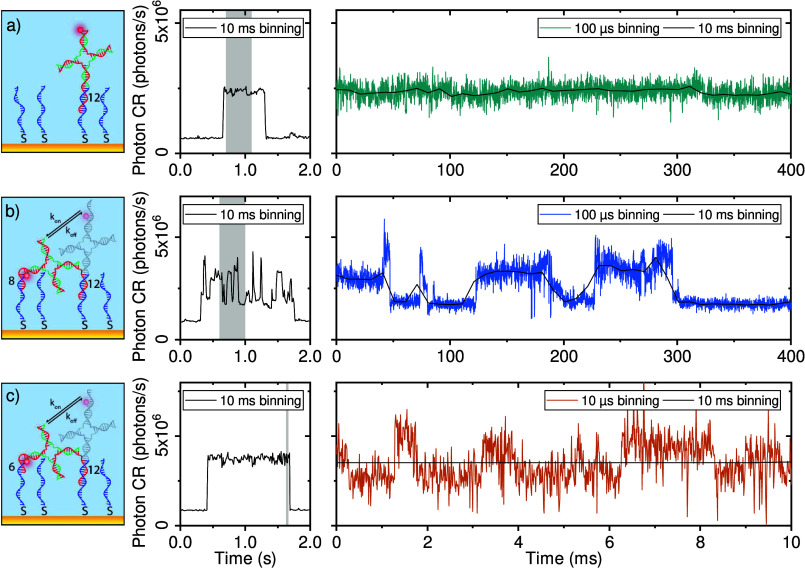
Intensity time traces of events recorded with three different HJ constructs: (a) HJ0, (b) HJ8, and (c) HJ6. Black traces are binned to 10 ms. The right column shows a zoom-in of the same data binned to 100 μs (a, b) or 10 μs (c). Gray areas highlight the parts enlarged in the right panels. Schematic illustrations of the three HJ constructs are shown to the left of the time traces. Note that typical event intensities are somewhat lower than for the simple monovalent ligands (Figure 3) due to the larger spacing between the dye and the AuNR.

For HJ0 the dye’s time-averaged distance to the AuNR is (nearly) constant (also see SI sections S6 and S7) which is reflected in a stable plasmon-enhanced fluorescence intensity even on microsecond time scales (Figure 4a). In contrast, HJ8 and HJ6 exhibit signal dynamics on microsecond time scales. Note that microsecond resolution is critical to reveal these dynamics because binning times of 10 ms (black lines in Figure 4, typical for regular single-molecule fluorescence) completely wash out the dynamics. For most HJs, the intensity modulations are around 20–50%, in good agreement with the expected distance modulation (see SI section S7).

We observe a subset of HJs that exhibit clear two-level behavior (as in Figure 4c), where state lifetimes can be extracted easily by change-point detection.^47^ The resulting distributions reveal a mean bound-state lifetime of a few milliseconds that follows a single-exponential behavior (see SI section S9). This indicates that the HJ can access one binding site with a well-defined rate constant and associated photon CR. The majority of events, however, exhibit complex multistate dynamics because multiple binding sites are accessible on the particle surface that each result in a different photon CR due to the inhomogeneous near-field around the particle (as in Figure 4b and SI section S8).

To enable quantification of the complex dynamics, we computed the autocorrelation function (ACF) of individual binding events; see Figure 5a and SI sections S8 and S10. This approach is analogous to fluorescence correlation spectroscopy (FCS), but the bright signals do not require averaging over many events: we directly extract the dynamics for each single molecule individually. We fitted the ACFs using a stretched exponential starting from 40 μs to eliminate the effect of the low-amplitude correlation at around 10 μs, which we attribute to residual blinking.

**Figure 5:**
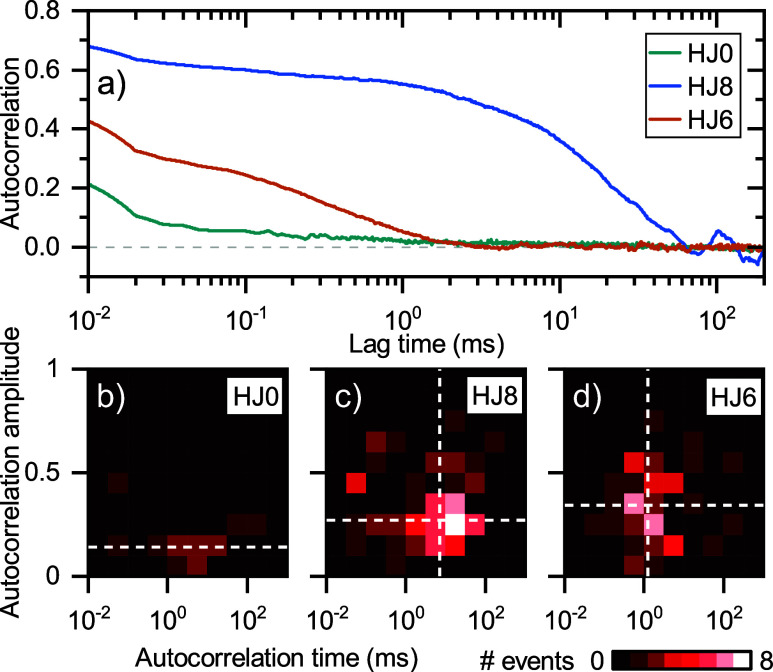
(a) Single-molecule autocorrelation functions computed from the time traces shown in Figure 4, based on 10 μs binning. (b–d) Density maps of the fitted characteristic time for each single molecule versus amplitude of the ACFs computed from all individual events of the different HJs. White dashed lines indicate the median autocorrelation time and amplitude of all of the molecules.

As expected, the ACF of HJ0 has a low correlation amplitude due to the absence of intramolecular dynamics. The characteristic ACF time scales for the selected HJ8- and HJ6-events are on the order of 10 ms and a few hundred μs, respectively, reflecting the fast intramolecular dynamics of HJ6. In Figure 5b–d we show density maps of the characteristic time versus the ACF amplitude across all individual events. The median characteristic times for the HJ8 and HJ6 are 7.0 and 1.2 ms, in agreement with the reduction in bound-state lifetime for 6 and 8 nt monovalent ligands in Figure 3c. The characteristic times are broadly distributed for both HJ8 and HJ6. This is attributed to a heterogeneous receptor density on the particle surface that results in a varying number and accessibility of nearby receptors for the low-affinity site. For HJ8 we find a weak correlation between the total bound-state lifetime (event duration) and the characteristic time in the ACF, indicating that the overall avidity of the complex is slightly enhanced by the 8 nt binding site. The ability to directly observe fast intramolecular dynamics and its heterogeneity is unique and will enable the rational design of multivalent substrates^48^ and the characterization of multivalent interactions on heterogeneous surfaces such as lipid bilayers.^18,49^

By engineering the excitation and decay rates of plasmon-coupled fluorophores, we achieved a photon CR of >10^7^ photons/s for monomeric nanoanatennas. We applied the emerging capabilities to two model systems, namely mono- and multivalent complexes. We resolve molecular dynamics on time scales of 1–10 μs with high signal-to-noise ratio, demonstrating the detection of transient encounters and monovalent binding, as well as intramolecular dynamics in multivalent constructs. The high signal to noise ratio of the approach eliminates the need for averaging and thereby reveals new insights into heterogeneity in microsecond molecular processes. Importantly, the technique is simple, requiring only basic sample preparation steps and a standard fluorescence microscope. We envision that this method can be used to study the microsecond dynamics of enzymes, IDPs, or even more complex biomolecular systems at the single-molecule level in real time. This opens the door to mechanistic studies of conformationally controlled biomolecular processes via a single-molecule approach in the relevant but previously inaccessible microsecond regime.

## Supporting Information Available

Materials and methods, BEM simulation of fluorescence enhancement, optimization of surface chemistry and buffer conditions, white light spectroscopy of individual AuNRs, more examples of time traces and bright time distributions for monovalent ligands, Holliday junction structure, DNA length estimates, more examples of HJ time traces and corresponding ACFs, HJ state lifetime analysis by change-point detection, and discussion of the autocorrelation analysis (PDF)
