# Supplementary material for: Rate-Engineered Plasmon-Enhanced Fluorescence for Real-Time Microsecond Dynamics of Single Biomolecules
Source: Nano Lett. 2024 Sep 9;24(37):11641–7. doi: 10.1021/acs.nanolett.4c03220 (PMC11421078; doi:10.1021/acs.nanolett.4c03220)
Supplement: Supplementary file 1 — nl4c03220_si_001.pdf [file nl4c03220_si_001.pdf]

Supplementary Information for:

Rate-engineered plasmon-enhanced fluorescence for real-time microsecond dynamics of single biomolecules

Sjoerd W. Nooteboom[1,2], Kasper R. Okholm[3,4], Vincenzo Lamberti[1,2], Bas Oomen[1,2], Duncan S. Sutherland[3,4], and Peter Zijlstra[1,2]

[1]Department of Applied Physics and Science Education, Eindhoven University of Technology, 5600 MB Eindhoven, The Netherlands

[2] Institute for Complex Molecular Systems, Eindhoven University of Technology, 5600 MB Eindhoven, The Netherlands

[3]Interdisciplinary Nanoscience Center, Aarhus University, 8000 Aarhus C, Denmark

[4]The Centre for Cellular Signal Patterns (CELLPAT), 8000 Aarhus C, Denmark

Contents

S1. Materials and methods 2
S2. Engineering singlet excitation and decay rate by BEM simulations 5
S3. Engineering triplet decay rate by surface chemistry and buffer conditions 8
S4. Single-particle spectroscopy 9
S5. More examples of timetraces and bright time distributions for monovalent ligands 10
S6. Holliday junction structure 13
S7. DNA end-to-end distance 14
S8. More examples of HJ timetraces and corresponding ACFs 15
S9. HJ state lifetime analysis by change-point detection 19
S10. Discussion of the autocorrelation analysis 20
References 20

S1. Materials and methods

Sample preparation. Microscope coverslips (thickness #1.5, Epredia) were cleaned by sonication in methanol for 15 min, then dried by nitrogen flow. The coverslips were plasma-treated for 1 min to render the surface hydrophilic. They were then immersed in a 5 vol. % solution of 3-mercaptopropyltrimethoxysilane (MPTMS) in ethanol for 3 min to create a monolayer of thiols by silanization, followed by rinsing with ethanol and drying by nitrogen flow.

Suspensions of AuNRs of different sizes (A12-10-650-CTAB, A12-25-650-CTAB, A12-40-650-CTAB, A12-70-650-CTAB, NanoPartz) were centrifuged at 10k rpm for 3 min and the supernatant replaced by a 1 mM solution of cetyltrimethylammonium bromide (CTAB) in distilled water. The resulting suspension was spin coated onto the coverslips, which were then rinsed with methanol, phosphate buffered saline (PBS) and distilled water to remove excess CTAB and unbound AuNRs, and dried by nitrogen flow.

Stick-on flow cells (Grace Bio-Labs) were attached to the AuNR-containing glass slides. A solution of 5 µM thiolated ssDNA (2-100% receptor strands, the remaining part spacer strands; Integrated DNA Technologies) and 1 mM tris(2-carboxyethyl)phosphine hydrochloride (TCEP, Merck) in citrate buffer (10 mM, pH 3, 1 M NaCl) was injected and incubated for ~2 h to form a ssDNA monolayer on the AuNRs [1,2]. All DNA sequences can be found in Supplementary Table S1 below. Afterwards, the flow cells were rinsed with 200 µL PBS and 200 µL buffer B (5 mM Tris-HCl, 10 mM $MgCl_2$, 1 mM EDTA, pH 8.0, filtered) and stored in the fridge for 1-10 days before use.

Scattering electron microscopy (SEM) measurements were performed to confirm the average particle size with the supplier's specification (see Figure S1). In addition we performed single-particle scattering spectroscopy (see methods below) to extract the wavelength and linewidth of the longitudinal SPR.

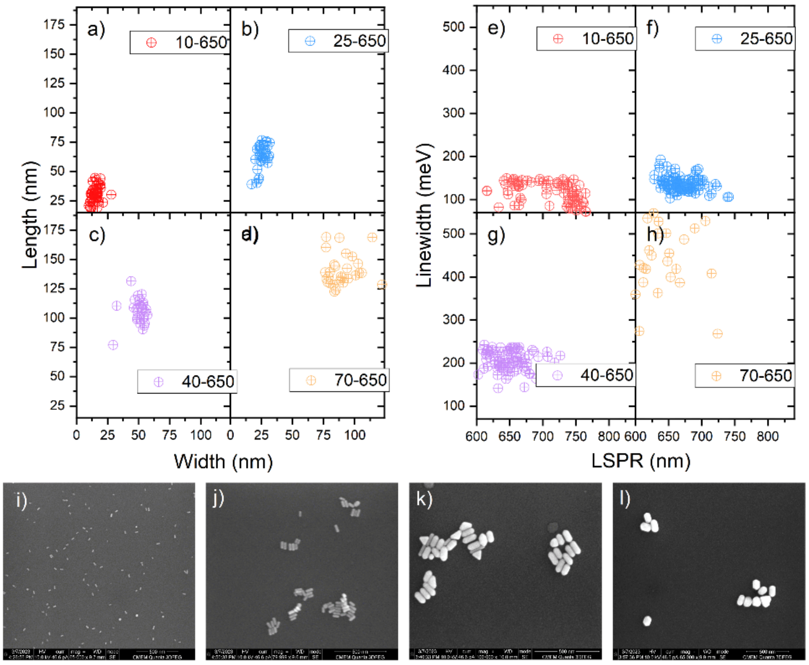

Figure S1. Dimensions and optical properties of the used nanoparticles with corresponding SEM snapshots. a-d) Distribution of length and width of the used rods obtained from the SEM images. e-h) longitudinal surface plasmon resonance wavelength and linewidth obtained from single-particle scattering spectroscopy of particles immobilized on glass. Legends refer to particles with the notation "diameter-LSPR (nm)" as indicated by the supplier. i-k) SEM snapshot of the stock nanoparticles sorted by size 10,25,40, and 70 nm width from i) to k).

Table S1. Sequences of the DNA oligos used in this work. Complementary parts for receptor-ligand binding are shown in red.

Strand name	Sequence
Receptor strand	5’-thiol-CAT CAT CAT ACG CTT CCA ATA ATA CAT CTA-3’
Spacer strand	5’-thiol-ACG CTA TCA G-3’
Monovalent ligand Atto655 9nt	5’-C TAG ATG TAT-Atto655-3’
Monovalent ligand Atto643 9nt	5’-Atto643-TAG ATG TAT-3’
Monovalent ligand Atto643 8nt	5’-Atto643-TAG ATG TA-3’
Monovalent ligand Atto643 7nt	5’-Atto643-TAG ATG T-3’
Monovalent ligand Atto643 6nt	5’-Atto643-TAG ATG-3’
Monovalent ligand Atto643 5nt	5’-Atto643-TAG AT-3’
Noncomplementary ligand	5’-Atto643-ATA CAA ACA ACA A-3’
R-Atto643	5’-Atto 643-CCC ACC GCT CGG CTC AAC TGG G-3’
X 12nt	5’-CCC AGT TGA GCG CTT GCT AGG GC TAG ATG TAT TAT-3’
B	5’-CCC TAG CAA GCC GCT GCT ACG G-3’
H	5’-CCG TAG CAG CGC GAG CGG TGG G -3’
H 8nt	5’-CCG TAG CAG CGC GAG CGG TGG G CTAG ATG TA-3’
H 6nt	5’-CCG TAG CAG CGC GAG CGG TGG G C TAG ATG-3’

Single-AuNR dark-field spectroscopy Single-AuNR scattering spectra were measured by objective-type total internal reflection microscopy on an inverted wide-field microscope (Nikon Ti2). The sample was illuminated by a collimated SuperK COMPACT supercontinuum laser (NKT Photonics) through an oil-immersion 1.49 NA objective. The excitation wavelength was tuned across the visible spectrum by an AOTF (SuperK Varia) with a bandwidth of 10 nm. A beam block was inserted into the objective's back aperture to block the direct reflection, while allowing the scattered light to pass through and reach an EMCCD camera (Andor iXon Ultra DU-888). The nanoparticle's point spread function (PSF) was fitted with a 2D Gaussian function to obtain the scattered intensity at each wavelength. The single-particle scattering spectrum was fitted with a Lorentzian (see Supplementary Fig. S6 below). AuNRs with an abnormally broad spectrum or a poor fit were excluded from the analysis.

Widefield fluorescence microscopy DNA ligands with Atto655 (Eurofins Genomics) were diluted to concentrations between 0.2-1 nM in buffer B and flushed into the sample chamber. The sample was illuminated by a 637 nm excitation laser (OBIS FP 637LX, Coherent). A quarter waveplate (Thorlabs) was used to achieve circular polarization and the sample was illuminated in epi mode to eliminate the effect of different AuNR orientations. The laser power density in the center of the field of view was approximately 10^8 W/m^2. The reflected laser light was suppressed by combining a dichroic mirror (ZT640rdc, Chroma), a notch filter (ZET635NF, Chroma), and a long-pass filter (FELH0650, Thorlabs) and the fluorescence signal was collected on the same camera used for the scattering spectra with a 50 ms exposure time.

Fluorescence timetraces were constructed by summing the pixel values of 7x7 regions of interest (ROIs) around the AuNRs, and converted to photons/s by dividing by the camera sensitivity (in A/D counts per photon, provided by the manufacturer) and exposure time. To obtain the PCR from the fluorescence events in the timetraces, the background due to the nanoparticle's one-photon photoluminescence was first removed by a moving median filter. Bursts were then identified by application of a threshold. Events with a maximum of 2 dark frames between them were merged. For

the nonenhanced PCR, the same procedure was repeated on ROIs near the center of the field of view without AuNRs in them and the highest bursts were averaged over ~100 ROIs.

Microsecond single-molecule dynamics Trolox (Merck) was dissolved in ethanol at a concentration of 160 mM. It was then diluted to 2 mM in imaging buffer (5 mM Tris-HCl, 300 mM NaCl, 1 mM EDTA, pH 8.0, filtered) and exposed to UV light for 15 min to form Trolox quinone. DNA ligands with Atto643 (Eurofins) were diluted to 1.5 nM in the Trolox-containing buffer and flushed into the sample chambers. A separate sample was used for every ligand to avoid signal contamination by ligands of different lengths. The excitation and detection paths were the same as in the widefield experiment except for the use of a broad band-pass filter (ET706/95m, Chroma) instead of a long-pass filter, and instead of the camera a SPAD (SPCM-AQRH-15, Excelitas, QY 70% at 700 nm) was used in combination with a TimeHarp 260 NANO Dual time tagger (PicoQuant). The signal from a single AuNR was projected onto the SPAD and photon arrival times were placed into bins of the desired duration.

Quantification of blinking dynamics Timetraces with 10 µs binning time were processed by the same event detection algorithm described above, allowing a maximum of 100 ms dark time for event merging. Events with a duration >50 ms and a PCR >$4x10^6$ photons/s were analyzed for blinking. The distribution of PCR values within an event was fitted to a double Gaussian (one for the on state and one for the off state). A threshold was set based on the minimum in this distribution and the duration of states above and below this threshold was analyzed.

Quantification of monovalent ligand dynamics Timetraces with 10 µs binning time were again processed by the event detection algorithm, where the allowed dark time was now set to 10, 5, 2, 1, 0.5, or 0.1 ms for 9nt, 8nt, 7nt, 6nt, 5nt, or noncomplementary ligands, respectively. To prevent artifacts due to separate short events being merged, events with a median PCR below threshold were discarded. The time between start and end of every event was then analyzed and processed into CDFs which were fitted with a double exponential.

Quantification of multivalent ligand dynamics The HJ strand carrying Atto643 was purchased from Eurofins; all other HJ strands were purchased from Integrated DNA Technologies. HJs were assembled by mixing the four strands (R, X, B, and H; see Supplementary Table S1) at equimolar concentrations in PBS, heating to 70°C, and cooling linearly to 4°C over 90 minutes. The HJ constructs were analyzed by 12% Native PAGE using 200V for 2h with 1xTBE as running buffer and appr. 0.5 pmol of HJ construct. The PAGE gel was made from 40% Accugel (National Diagnostics) and was stained with Sybr Gold (Invitrogen). Invitrogen UltraLow range DNA ladder was used as a reference. The gel was stained and imaged with an Amersham Typhoon laser scanner (see Supplementary Fig. S12).

Trolox was added to TAE buffer (40 mM Tris-HCl, 20 mM acetic acid, 1 mM EDTA) with 10 mM $MgCl_2$, in the same way as described above. The HJs were then diluted to 1 nM in this buffer. The solution was flushed into the sample chambers and the measurements were performed in the same way as those on monovalent ligands. Autocorrelation curves were produced by applying the Matlab function autocorr to the 10 µs binned timetraces, and fitted to a stretched exponential.

S2. Engineering singlet excitation and decay rate by BEM simulations

Simulations of AuNR plasmon resonances and fluorescence enhancement were performed with the boundary element method (BEM), using the MNPBEM17 toolbox [3]. AuNRs were approximated as cylinders capped by hemispheres. The dielectric function of gold was set to the values tabulated by Johnson & Christy [4]. The refractive index of the medium was set to 1.33 and the simulations neglected the effect of the substrate. The excitation wave with wavelength 637 nm was polarized along the AuNR long axis. Decay rate enhancements were computed across the dye's full emission spectrum, and combined with the intrinsic emission amplitudes following the approach of ref [5].

Figure S2 shows the meshing used for simulations of a 40x82 nm^2 AuNR. Excitation enhancement is calculated by exciting the AuNR with a plane wave, polarized along its long axis, and simulating the electric field around the AuNR. Then $k_{exc}/k_{exc}^0 = |E|^2/|E_0|^2$.

Radiative and total decay rate enhancements were computed directly with the BEM toolbox, which outputs the decay rates normalized to the dye's intrinsic radiative decay rate k_r^0. The rate enhancements as a function of frequency are $\xi_r(\omega) = k_r(\omega)/k_r^0$ and $\xi_{tot}(\omega) = k_{tot}(\omega)/k_r^0$. These enhancements were computed across the entire dye spectrum for better accuracy and then integrated over all frequencies:

$$k_r = k_r^0 \int f^0(\omega)\xi_r(\omega)d\omega$$

$$k_{nr} = k_{nr}^0 + k_r^0 \int f^0(\omega)[\xi_{tot}(\omega) - \xi_r(\omega)]\, d\omega$$

where k_{nr}^0 is the intrinsic nonradiative decay rate and $f^0(\omega)$ is the area-normalized intrinsic dye spectrum. The total PCR enhancement in the case without saturation is then:

$$\frac{PCR}{PCR_0} = \frac{k_{exc}}{k_{exc}^0} * \frac{k_r/(k_r + k_{nr})}{k_r^0/(k_r^0 + k_{nr}^0)}$$

Different dye positions along the AuNR circumference at 5 nm distance were evaluated and the position yielding the highest enhancement was selected to represent the best-case scenario. For each AuNR diameter, a range of lengths was evaluated and the optimum dimensions were identified. These optimum dimensions lead to the maximum photon CR (black line in Figure S3), and correspond to 10x24, 25x56, 40x82,and 70x114 nm^2 particles, respectively. The results are shown in Figure S3 for these AuNR dimensions.

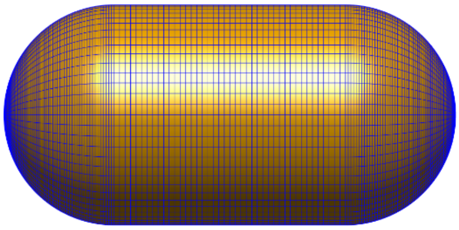

Figure S2. 40x82 nm^2 AuNR with meshing as used in the simulations.

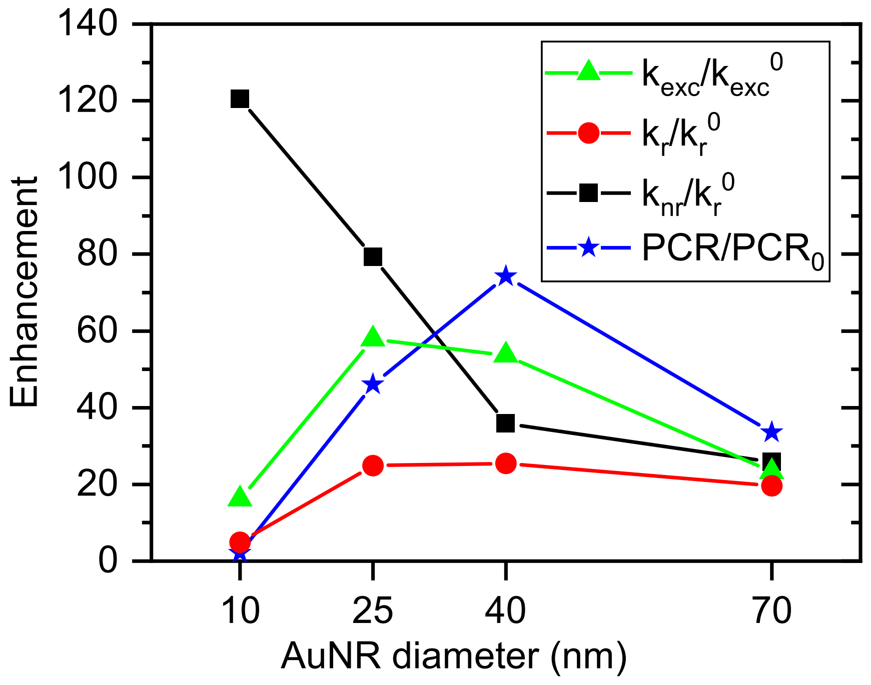

Figure S3. The enhancement of the most important quantities of Atto655 at the optimum binding position with 5 nm distance from the AuNR surface. For every diameter, the result of the AuNR length with the best PCR enhancement is shown (10x24, 25x56, 40x82,and 70x114 nm^2 particles).

In this size regime the higher radiative efficiency of larger nanoantennas is compensated for by a broader plasmon linewidth caused by radiation damping. This means that at or above saturation the PCR is nearly diameter-independent in our intermediate size regime. In the experiments however we do observe a dependence of the PCR on nanoantenna diameter, indicating that we operate just below saturation where the excitation rate is maximum for 40 nm AuNRs due to their high near-field enhancement and low nonradiative losses. Figure S4, Figure S5, and Figure S6 further show the spatial profiles of k_{exc}/k_{exc}^0, k_r/k_r^0, and PCR/PCR_0, respectively, around the 40x82 nm^2 AuNR.

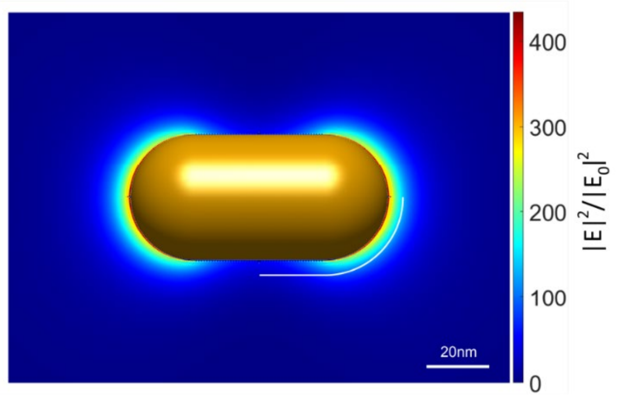

Figure S4. Excitation enhancement $|E|^2/|E_0|^2$ around a 40x82 nm^2 AuNR. The fringes near the surface are interpolation artifacts. The white line indicates a 5 nm distance from the AuNR surface, where the theoretical optimum enhancement is evaluated.

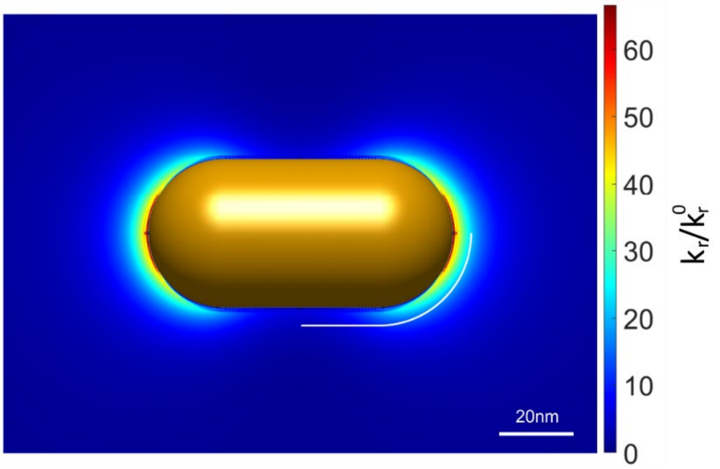

Figure S5. Radiative decay rate enhancement k_r/k_r^0 for Atto655 around a 40x82 nm^2 AuNR.

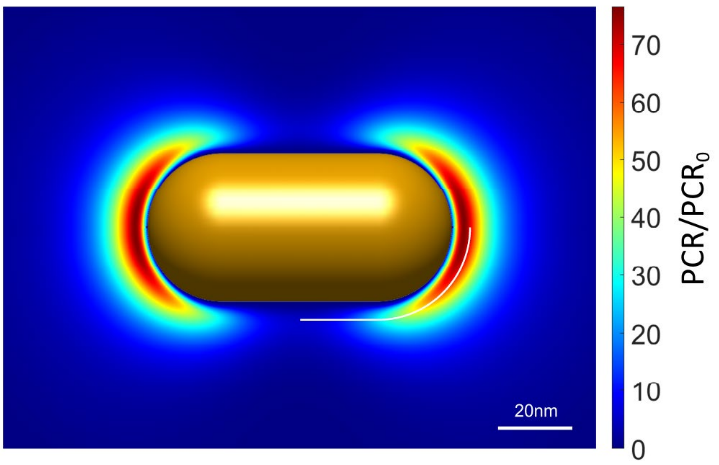

Figure S6. Photon count rate enhancement PCR/PCR_0 (without saturation) for Atto655 around a 40x82 nm^2 AuNR.

S3. Engineering triplet decay rate by surface chemistry and buffer conditions

The high PCR uniquely enables us to quantify microsecond blinking dynamics in real-time by projecting the signal from a single particle onto a SPAD that enables arbitrary time-binning. The middle panel in Figure 2 in the main text shows the start of a typical event for a 9nt ligand with an Atto655 label pointing toward the nanoparticle surface as in Figure 1b in the main text. Clear dark states are observed with durations ranging from 10 µs to >10 ms, while the dye spends on average 32% of the time in a dark state. We find better signal stability for an Atto643 label pointing outward into solution, possibly due to a better photostability of the fluorophore. Upon adding Trolox, a well-known triplet state quencher [6] the observed off-blinks become markedly shorter. To further reduce the duration of dark states we employed a mixed monolayer of ssDNA receptors and shorter ssDNA spacers to maximize the accessibility of the dye to solution-phase Trolox. In this case, off-blinks lasting longer than 200 µs are virtually eliminated, while the dye (on average) spends only 6% of its time in a dark state (see the right column in Figure S7).

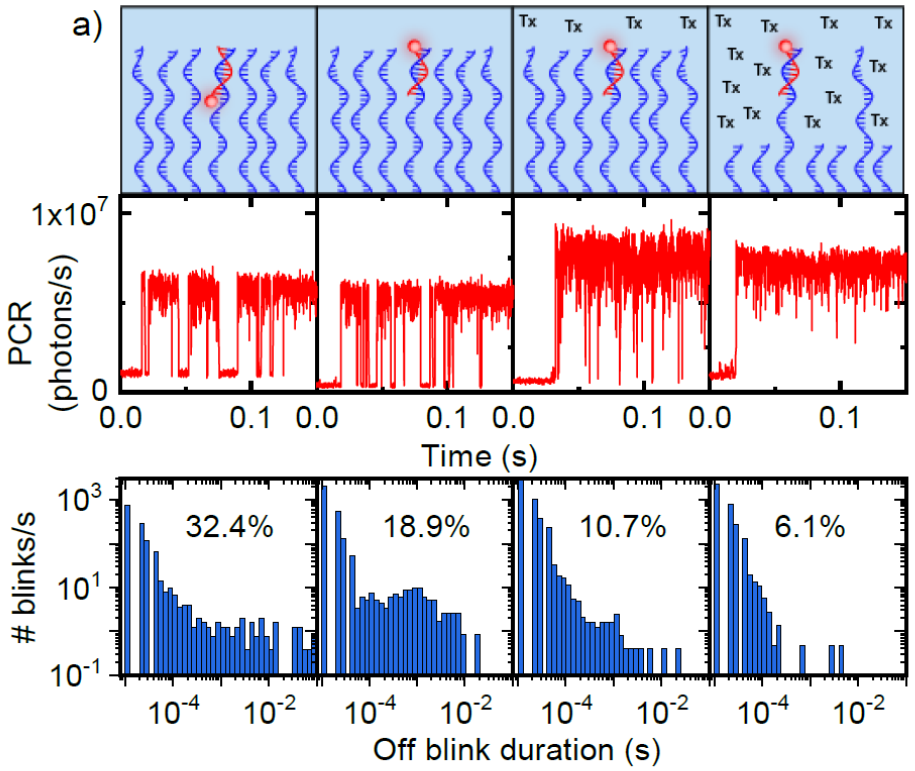

Figure S7. Top: schematic comparison of the DNA micro-environment corresponding to the results below: Atto655 inward; Atto643 outward; Atto643 with 2 mM Trolox (Tx); Atto643 with 2 mM Trolox in a mixed monolayer of ssDNA receptors and spacer strands. Middle: representative events with 100 µs binning time, illustrating the reduction of blinking. Bottom: the number of blinks per second as a function of off blink duration under the four different conditions, obtained from 10 µs binned timetraces. The percentages indicate the fraction of time the dye spends in the dark state.

S4. Single-particle spectroscopy

Figure S8 shows the setup and some example data of the white light spectroscopy as described in the Methods section.

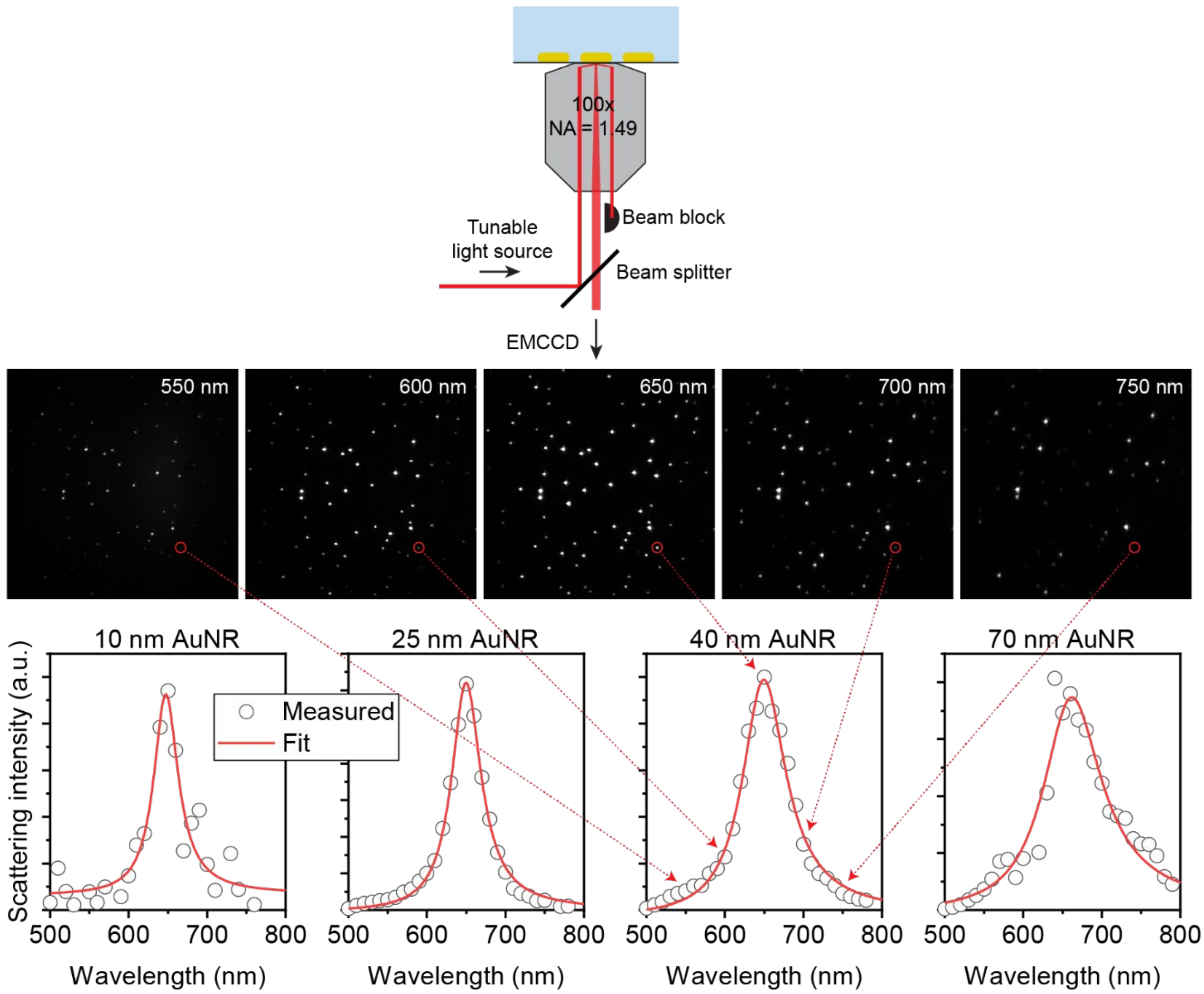

Figure S8. Top: schematic illustration of the setup for white light spectroscopy. Middle: the field of view with dozens of AuNRs at five different wavelengths. Bottom: example white light scattering spectra for typical AuNRs within each sample, fitted with a Lorentzian function.

S5. More examples of timetraces and bright time distributions for monovalent ligands

Figure S9, Figure S10, and Figure S11 show some example binding events for 9, 7, and 5nt ligands. 9 and 7nt are shown with 100 µs binning for clarity, whereas 5nt is shown with 10 µs binning. In all cases, the displayed events include both specific binding (relatively long) and diffusing ligands (very short). The bottom left 9nt event likely includes a diffusive event during a specific event, but this does not significantly impact the analysis.

Figure S12 shows the distribution of event lifetimes, similar to Figure 4a in the main text, but each panel shows results from several individual AuNRs with the same ligand. Some particle-to-particle heterogeneity can be observed in the lifetimes of the specific binding events, as captured by the boxplots in Figure 4b in the main text. On the other hand, the diffusive events all have fitted lifetimes of about 9 µs independent of the ligand length.

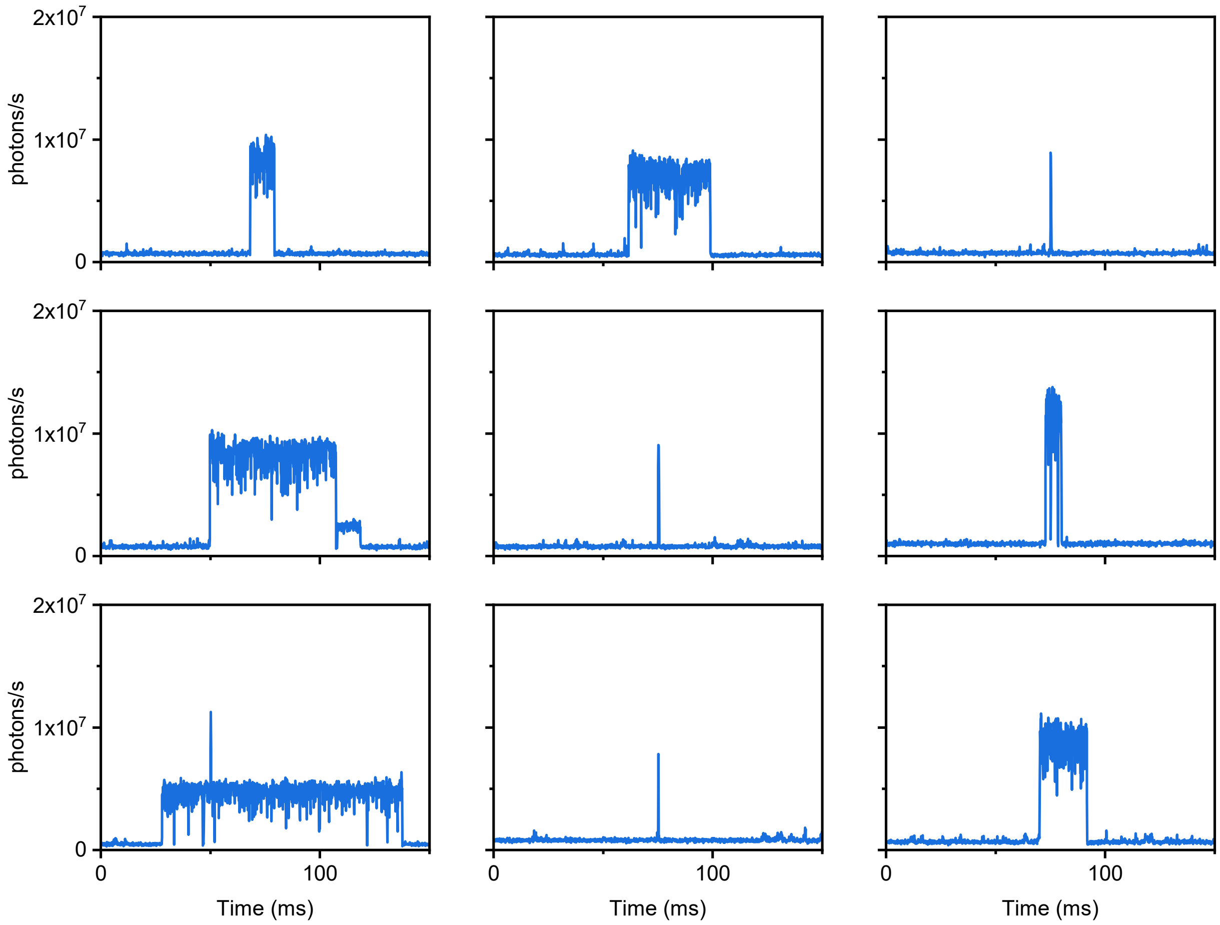

Figure S9. Events recorded with a 9nt ligand using 100 µs binning.

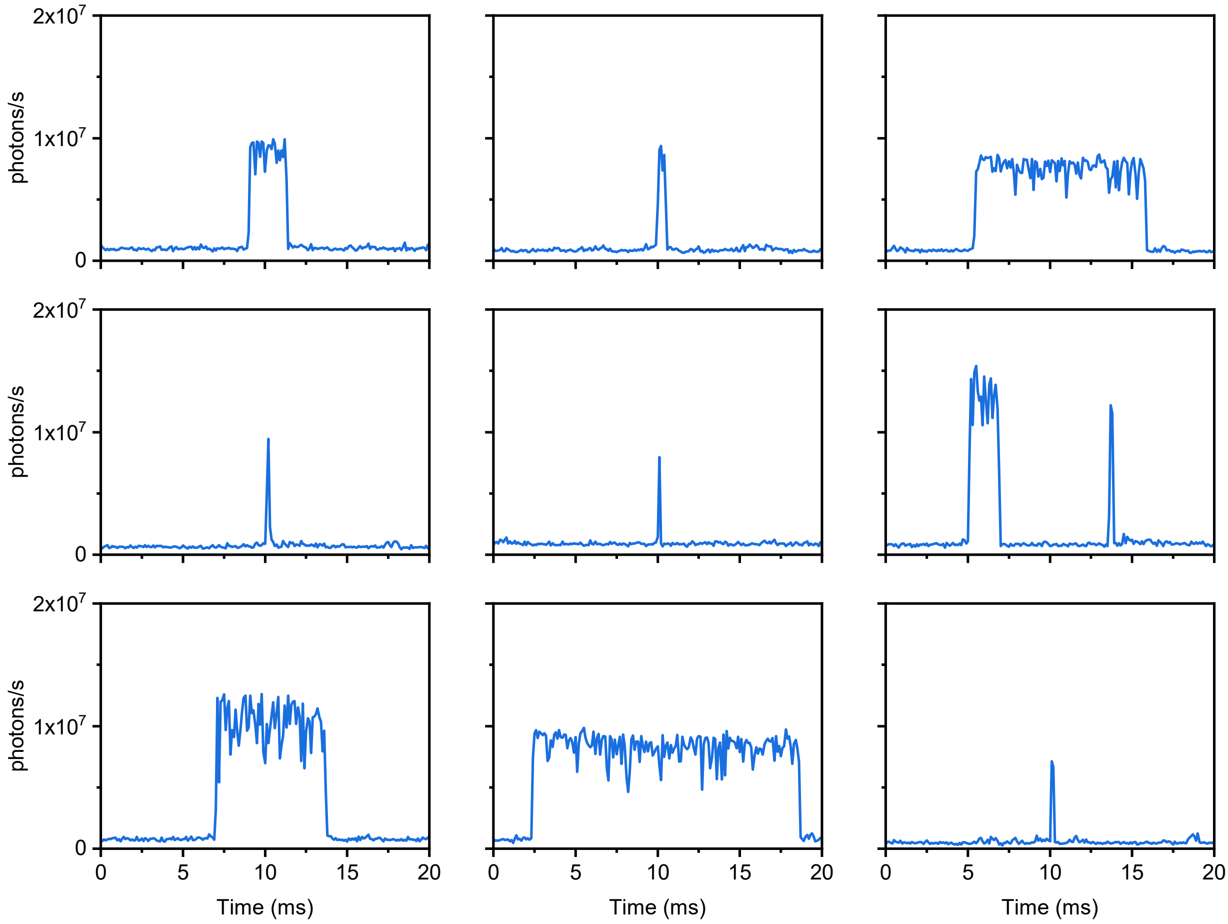

Figure S10. Events recorded with a 7nt ligand using 100 µs binning.

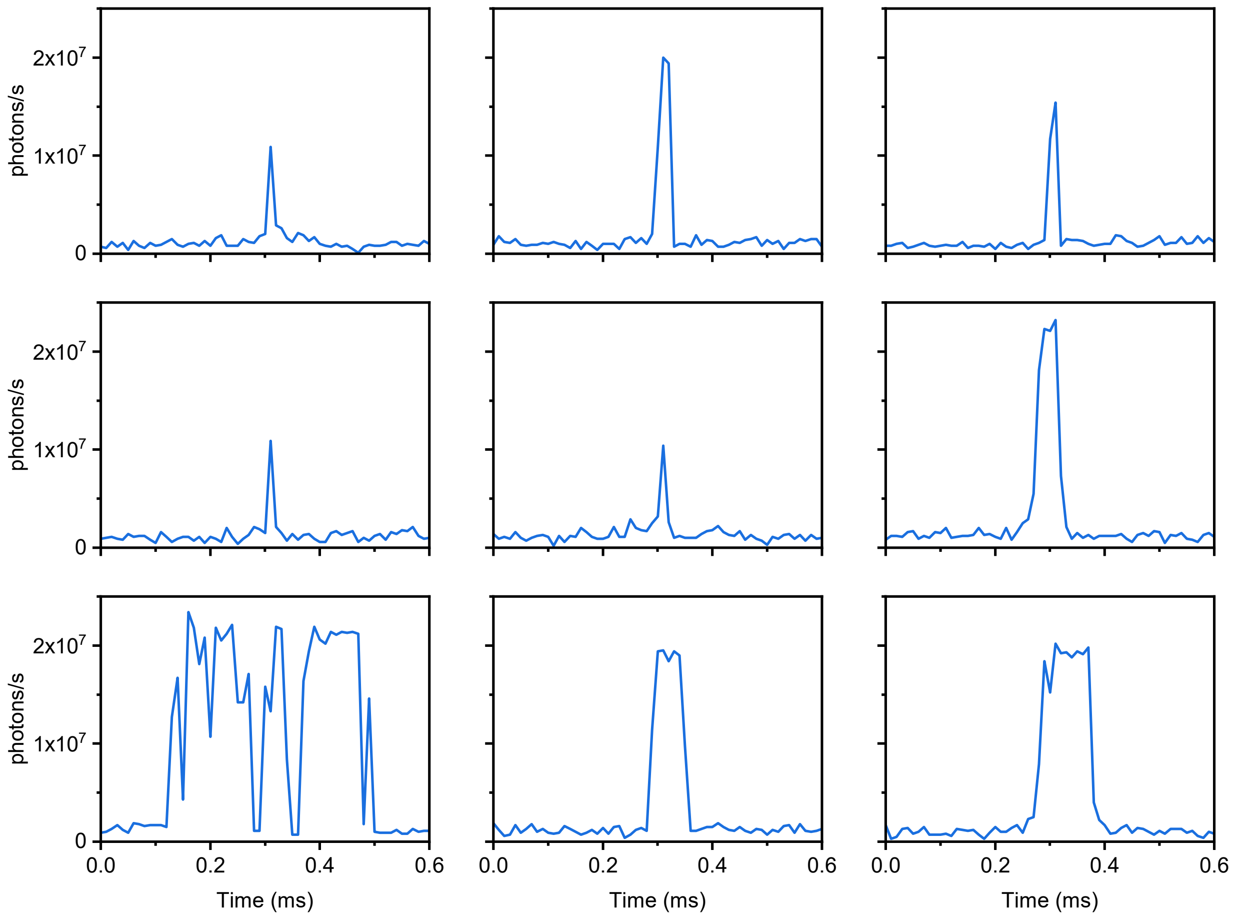

Figure S11. Events recorded with a 5nt ligand using 10 µs binning.

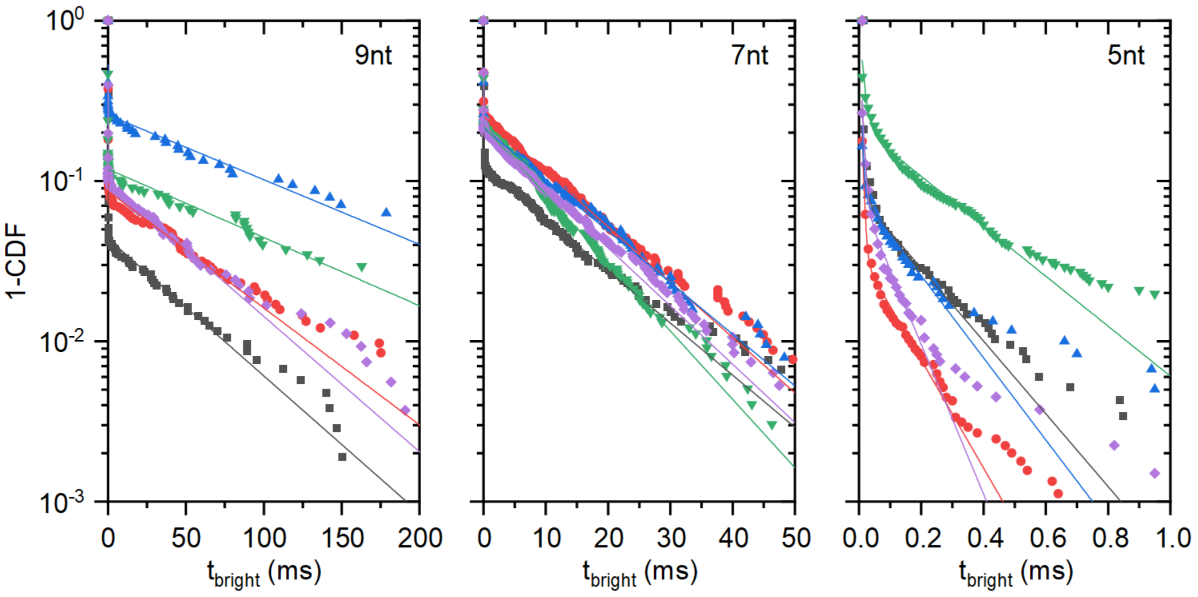

Figure S12. Binding time distributions recorded on some AuNRs with 9nt, 7nt, and 5nt ligands with double exponential fits.

S6. Holliday junction structure

The Holliday junction used in this work is referred to as Junction 7 in ref. [7]. The strands are labeled R, X, B, and H as shown in Figure S13 and Table S1. In the presence of salt, the HJ arms stack in two distinct isoforms and can transition between these two. It has been shown that Junction 7 co-exists in the two isoforms at almost equal ratios with a transition rate of about 10 s^{-1} [7] which is much slower than the observed dynamics in the HJ timetraces. These are therefore expected to only occur a few times during one binding event. For the HJ0, the 12nt extension and the position of the dye are chosen such that they are far apart in both isoforms (see Figure S13), to minimize the influence of isoform switching on the signal intensity and focus on multivalent binding instead.

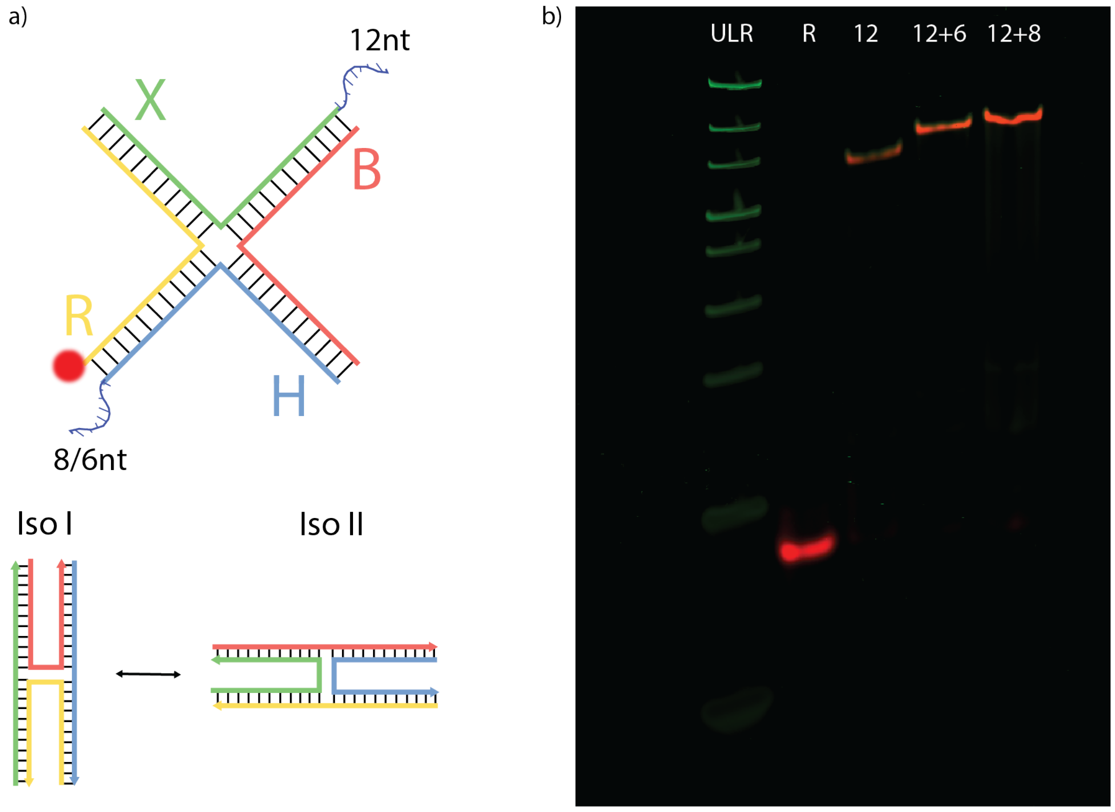

Figure S13. a) Sketch of the Holliday Junction and the stacking isoforms, showing the position of the dye and the sequence extensions for binding. b) 12% PAGE analysis of assembled HJs. From left to right: UltraLow range DNA ladder, single stranded HJ arm R carrying Atto643, HJ with a single 12nt extension, HJ with a 12nt and 6nt extension and finally a HJ with 12nt and 8nt extension. There is a slight shift in the HJ bands due to the extended arms.

S7. DNA end-to-end distance

To estimate the distance from the dye to the AuNR in different situations, we use the standard value of 0.34 nm/bp for double-stranded DNA (dsDNA). For single-stranded DNA (ssDNA), we use the expression [8]:

$$\langle R^2 \rangle = 2l_p R_{max} - 2l_p^2 \left(1 - \exp\left(-\frac{R_{max}}{l_p}\right)\right)$$

where $\langle R^2 \rangle$ is the time-averaged squared end-to-end distance of a polyelectrolyte, l_p is the persistence length, and R_{max} is the contour length, equal to the number of nt times 0.5 nm for ssDNA. For l_p we use the Debye length λ_D:

$$l_p = \lambda_D = \sqrt{\frac{\epsilon_r \epsilon_0 k_B T}{2000 e^2 N_A \sum_i c_i z_i^2}}$$

where ϵ_r is the relative permittivity of the medium, ϵ_0 is the permittivity of free space, $k_B T$ is the thermal energy, e is the charge of the electron, N_A is Avogadro's number, and c_i and z_i are the concentration and charge number of each type of ion in solution. At room temperature and for 10 mM $MgCl_2$ in water, the l_p value is 1.2 nm.

The resulting distance for the Atto655-labeled ligand (21nt of ssDNA separating the dye from the AuNR) is 4.7 nm. For the bivalent state of the HJ, we approximate the situation as just a 30nt ssDNA spacing, yielding 5.8 nm. The monovalent state is approximated as 18nt ssDNA (4.3 nm) combined with 34bp dsDNA (11.6 nm), which we combine according to the Pythagorean theorem to account for tilting of the HJ outside the receptor layer, to yield 12.3 nm. For enhancement estimations, all these values are rounded up to account for the presence of the C_6 linker between the thiol group and the DNA.

S8. More examples of HJ timetraces and corresponding ACFs

Figure S14, Figure S15, and Figure S16 show more examples of events with the HJ0, HJ8, and HJ6, respectively, similar to the examples in Figure 5 in the main text. The left columns show the entire events with 10 ms binning. The right columns show zoomed-ins parts of the left columns, where the HJ0 and HJ8 are binned to 100 µs for clarity and the HJ6 is binned to 10 µs.

Figure S17 shows the ACFs of all the events in Figure S14, Figure S15, and Figure S16. Each color corresponds to the event displayed in the same color. Like the ACFs in Figure 6a in the main text, the HJ0 events have much lower correlation amplitudes than the HJ8 and HJ6, while the characteristic times for the HJ8 are visibly longer than those of the HJ6.

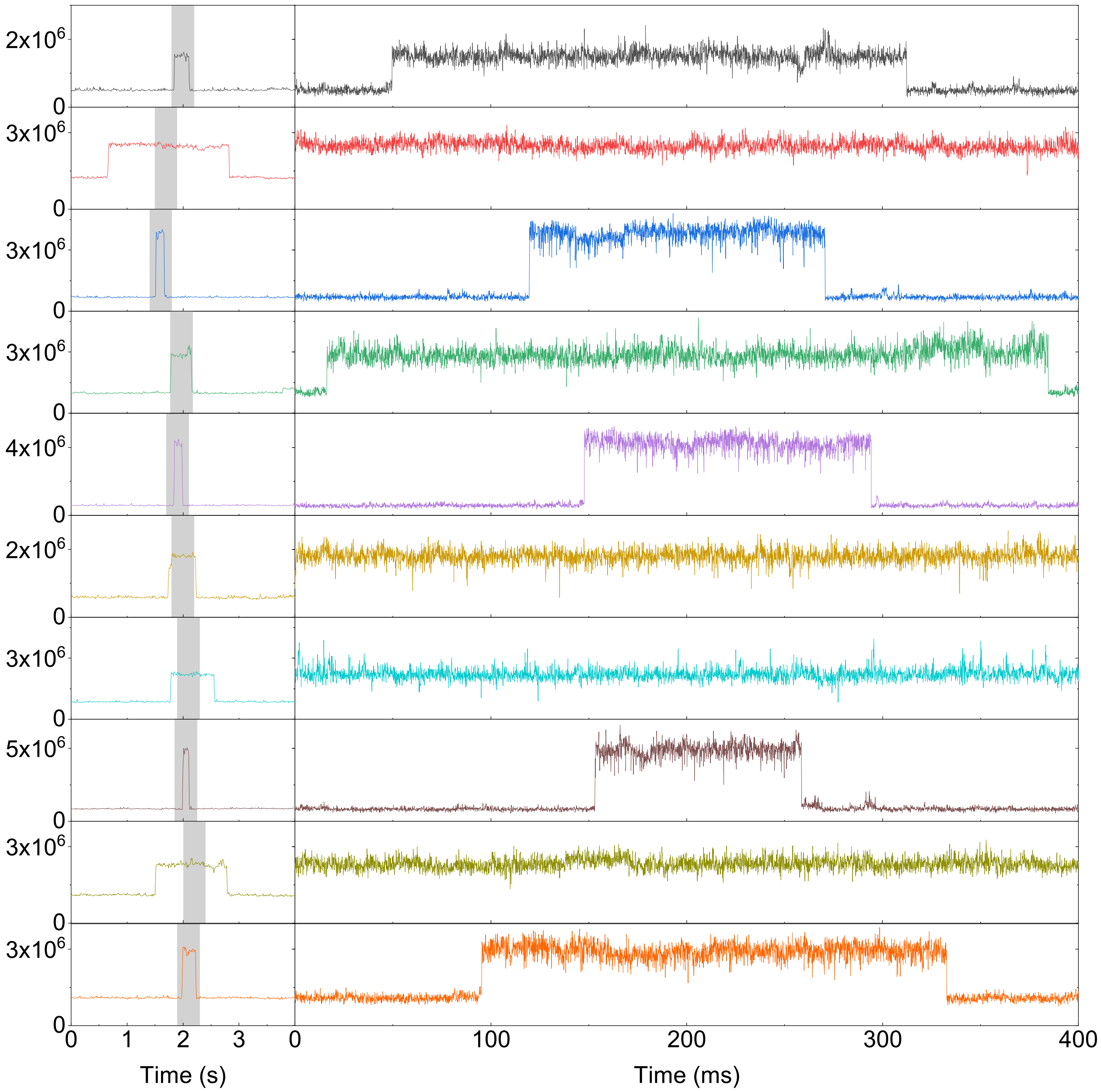

Figure S14. Example events of the HJ0 with 10 ms binning (left) and 100 µs binning (right). Gray bands indicate the parts that are zoomed into on the right. Note that the traces are scaled individually in the y direction for clarity.

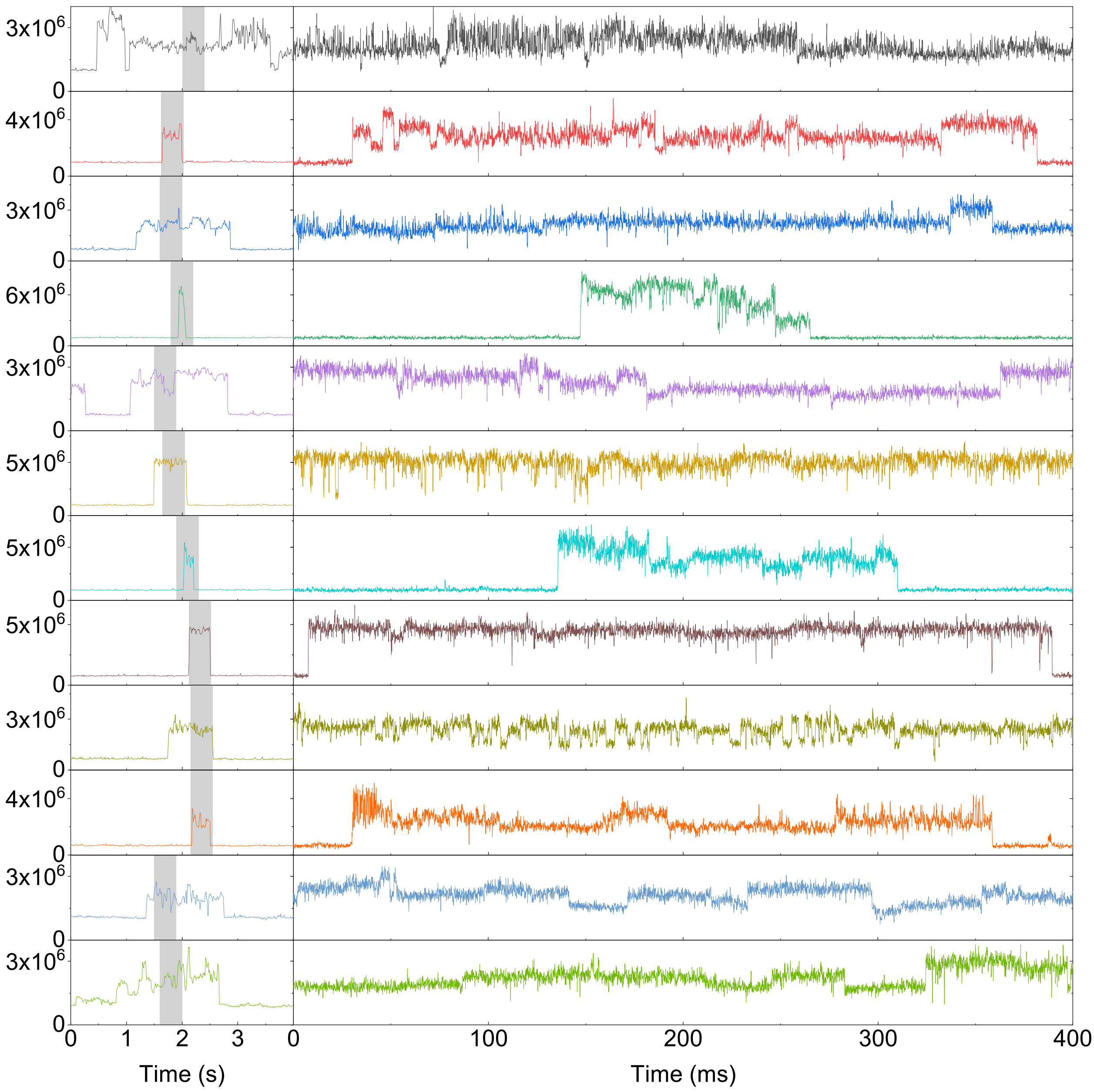

Figure S15. Example events of the HJ8 with 10 ms binning (left) and 100 µs binning (right). Gray bands indicate the parts that are zoomed into on the right. Note that the traces are scaled individually in the y direction for clarity.

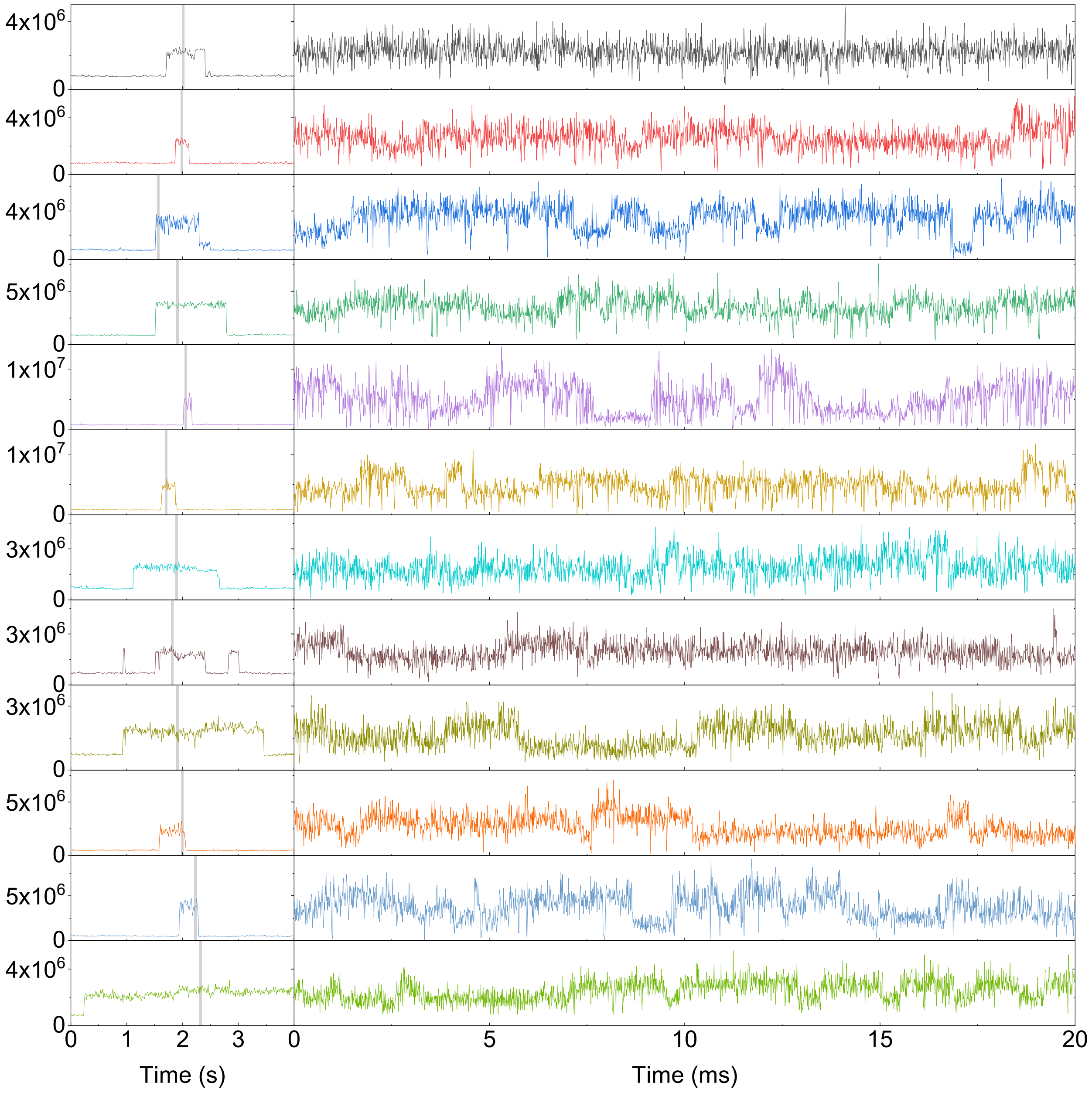

Figure S16. Example events of the HJ6 with 10 ms binning (left) and 10 μs binning (right). Gray bands indicate the parts that are zoomed into on the right. Note that the traces are scaled individually in the y direction for clarity.

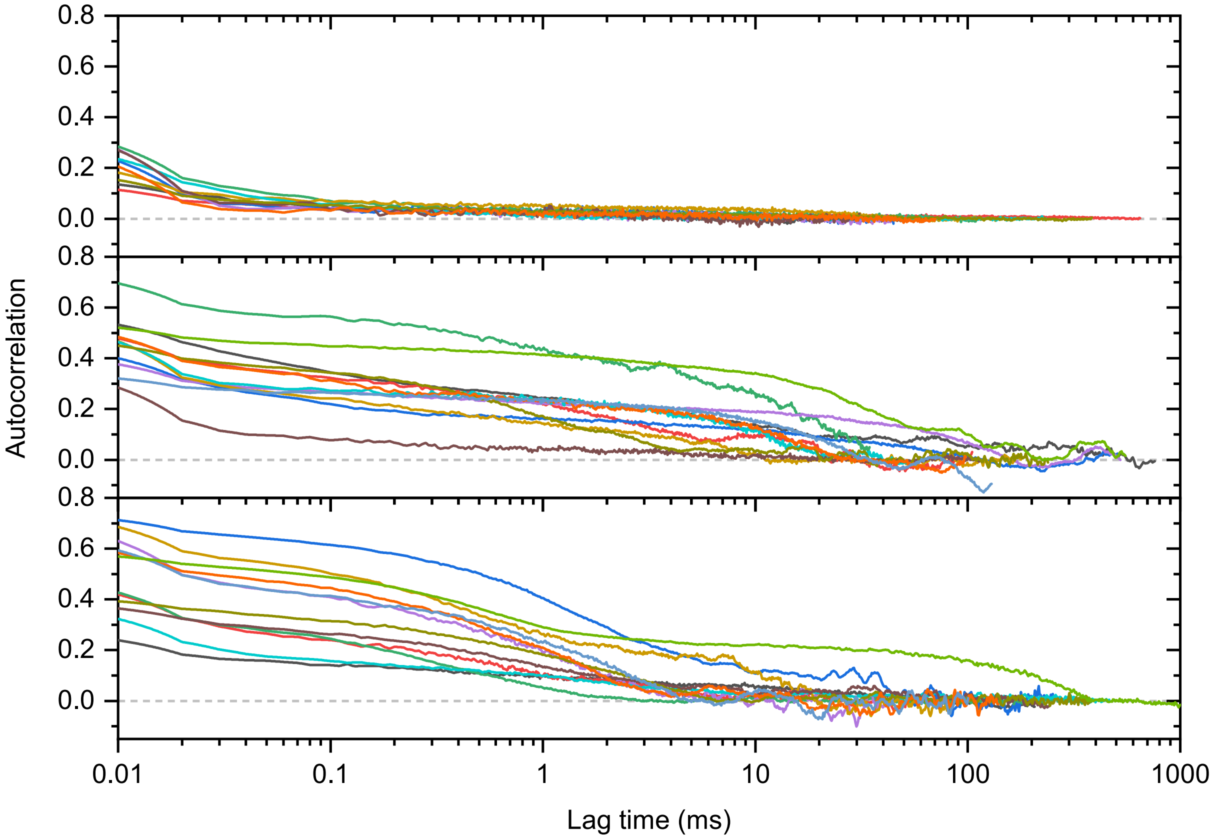

Figure S17. ACFs of the events displayed above (top: HJ0, middle: HJ8, bottom: HJ6), with each color corresponding to the event displayed in the same color.

S9. HJ state lifetime analysis by change-point detection

Figure S18 shows three example events of HJs with simple two-state behavior where change-point detection between monovalent and bivalent states can be applied and yields single exponential distributions of state lifetimes. However, these events only represent a small minority of events for which this is possible; therefore statistics across all events were instead obtained from ACF fitting.

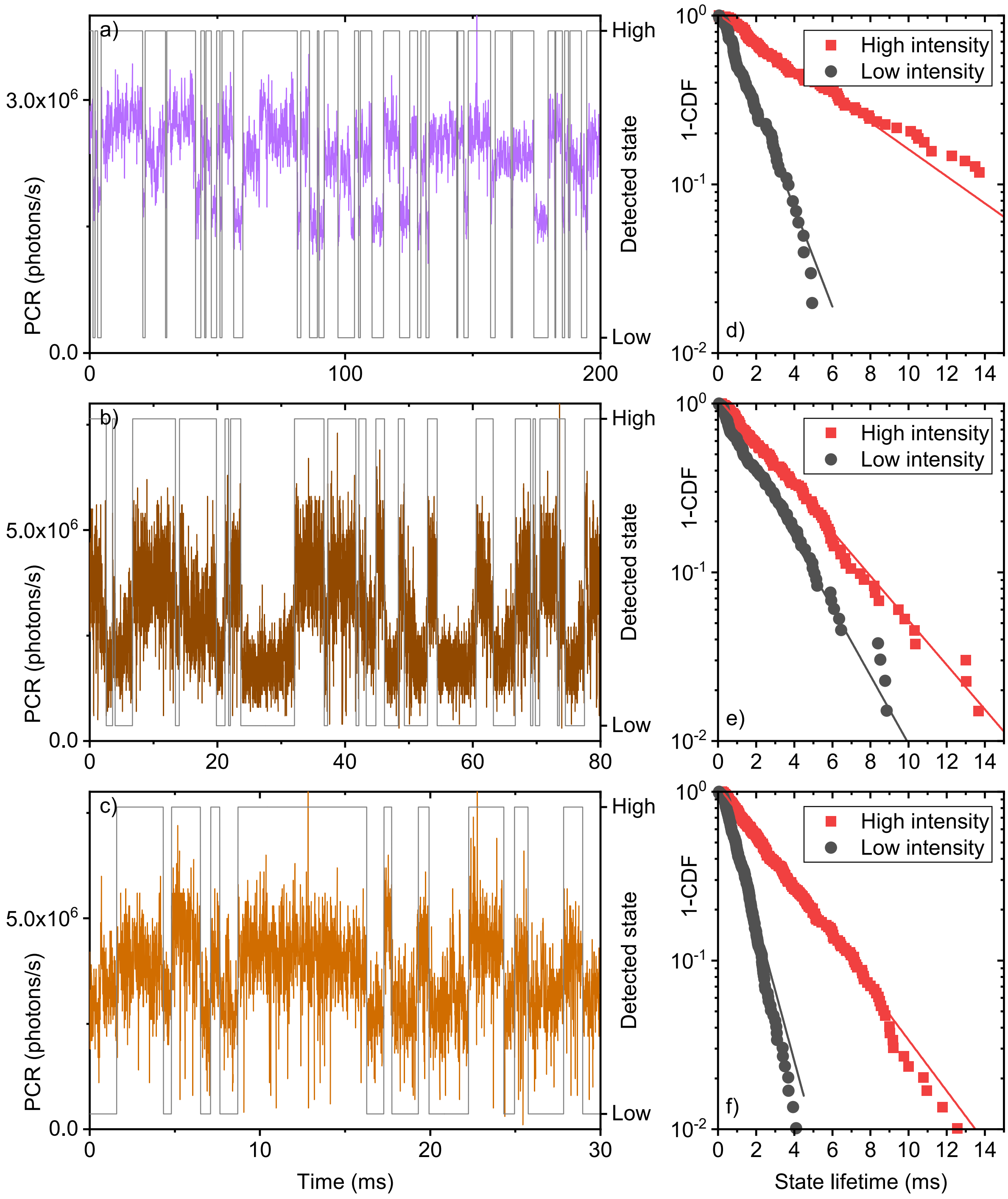

Figure S18. a-c) Timetraces of events with the HJ8 (a, 100 µs binning) and HJ6 (b-c, 10 µs binning), with the states determined by change-point detection overlaid in gray. d-f) The lifetime distributions of the high- and low-intensity states in the events, fitted to single exponentials.

S10. Discussion of the autocorrelation analysis

We constructed ACF curves of all the HJ events. This was done by extracting the intensity timetrace within each event with 10 µs binning and using the Matlab function `autocorr`. The ACFs were then interpolated to logarithmically spaced lag times in order to weigh each timescale equally for the fitting.

Because many events display dynamics on a mix of timescales, we fitted the ACFs using a stretched exponential ($y = \mathrm{A} * \exp\left[\left(-\frac{t}{\tau_{AC}}\right)^{\beta}\right]$), starting from 40 µs to avoid the influence of the ~10 µs blinking. Fits where the characteristic time τ_{AC} hit the lower limit of 0.01 ms or the amplitude A hit the upper limit of 1 were excluded.

The median autocorrelation times for the HJ8 and HJ6 (7.0 and 1.2 ms, respectively) are slightly lower than the median binding times of 35 and 2 ms, respectively, reported in Figure 4b in the main text for monovalent 8nt and 6nt ligands, respectively. This could be due to the fact that the characteristic AC time is determined by both binding and unbinding, $\tau_{AC} = \left[\tau_{on}^{-1} + \tau_{off}^{-1}\right]^{-1}$, and is hence shorter than just τ_{on} [9].

Furthermore, a few fits for HJ0 and HJ8 have relatively high A and low τ_{AC} values. Detailed investigation of these fits indicates that these may be artefacts of the fit, because the actual ACFs in these cases have low amplitudes, suggesting no dynamics within the event. In such cases, a stretched exponential with high A, low τ_{AC}, and low β is the best fit mathematically but not very meaningful physically.

References

1. M. Horáček, D. J. Engels, and P. Zijlstra, "Dynamic single-molecule counting for the quantification and optimization of nanoparticle functionalization protocols," Nanoscale **12**, 4128–4136 (2020).
2. S. Dey, R. Rivas-Barbosa, F. Sciortino, E. Zaccarelli, and P. Zijlstra, "Biomolecular interactions on densely coated nanoparticles: a single-molecule perspective," Nanoscale 10.1039.D3NR06140J (2024).
3. U. Hohenester and A. Trügler, "MNPBEM - A Matlab toolbox for the simulation of plasmonic nanoparticles," Comput. Phys. Commun. **183**, 370–381 (2012).
4. P. B. Johnson and R. W. Christy, "Optical Constants of the Noble Metals," Phys. Rev. B **6**, 10 (1972).
5. M. Ringler, A. Schwemer, M. Wunderlich, A. Nichtl, K. Kürzinger, T. A. Klar, and J. Feldmann, "Shaping emission spectra of fluorescent molecules with single plasmonic nanoresonators," Phys. Rev. Lett. **100**, 1–4 (2008).
6. T. Cordes, J. Vogelsang, and P. Tinnefeld, "On the mechanism of trolox as antiblinking and antibleaching reagent," J. Am. Chem. Soc. **131**, 5018–5019 (2009).
7. S. A. McKinney, A.-C. Déclais, D. M. J. Lilley, and T. Ha, "Structural dynamics of individual Holliday junctions," Nat. Struct. Biol. **10**, 93–97 (2003).
8. M. Rubinstein and R. H. Colby, *Polymer Physics* (Oxford University Press, 2003).
9. J. Mücksch, P. Blumhardt, M. T. Strauss, E. P. Petrov, R. Jungmann, and P. Schwille, "Quantifying Reversible Surface Binding via Surface-Integrated Fluorescence Correlation Spectroscopy," Nano Lett. **18**, 3185–3192 (2018).
